# TFIIS-Dependent Non-coding Transcription Regulates Developmental Genome Rearrangements

**DOI:** 10.1371/journal.pgen.1005383

**Published:** 2015-07-15

**Authors:** Kamila Maliszewska-Olejniczak, Julita Gruchota, Robert Gromadka, Cyril Denby Wilkes, Olivier Arnaiz, Nathalie Mathy, Sandra Duharcourt, Mireille Bétermier, Jacek K. Nowak

**Affiliations:** 1 Institute of Biochemistry and Biophysics, PAS, Warsaw, Poland; 2 Institute for Integrative Biology of the Cell (I2BC), CNRS, CEA, Université Paris Sud, Gif-sur-Yvette, France; 3 Institut Jacques Monod, CNRS, UMR 7592, Université Paris Diderot, Sorbonne Paris Cité, Paris, France; Washington University in St. Louis, UNITED STATES

## Abstract

Because of their nuclear dimorphism, ciliates provide a unique opportunity to study the role of non-coding RNAs (ncRNAs) in the communication between germline and somatic lineages. In these unicellular eukaryotes, a new somatic nucleus develops at each sexual cycle from a copy of the zygotic (germline) nucleus, while the old somatic nucleus degenerates. In the ciliate *Paramecium tetraurelia*, the genome is massively rearranged during this process through the reproducible elimination of repeated sequences and the precise excision of over 45,000 short, single-copy Internal Eliminated Sequences (IESs). Different types of ncRNAs resulting from genome-wide transcription were shown to be involved in the epigenetic regulation of genome rearrangements. To understand how ncRNAs are produced from the entire genome, we have focused on a homolog of the TFIIS elongation factor, which regulates RNA polymerase II transcriptional pausing. Six TFIIS-paralogs, representing four distinct families, can be found in *P*. *tetraurelia* genome. Using RNA interference, we showed that *TFIIS4*, which encodes a development-specific TFIIS protein, is essential for the formation of a functional somatic genome. Molecular analyses and high-throughput DNA sequencing upon *TFIIS4* RNAi demonstrated that TFIIS4 is involved in all kinds of genome rearrangements, including excision of ~48% of IESs. Localization of a GFP-TFIIS4 fusion revealed that TFIIS4 appears specifically in the new somatic nucleus at an early developmental stage, before IES excision. RT-PCR experiments showed that TFIIS4 is necessary for the synthesis of IES-containing non-coding transcripts. We propose that these IES+ transcripts originate from the developing somatic nucleus and serve as pairing substrates for germline-specific short RNAs that target elimination of their homologous sequences. Our study, therefore, connects the onset of zygotic non coding transcription to the control of genome plasticity in *Paramecium*, and establishes for the first time a specific role of TFIIS in non-coding transcription in eukaryotes.

## Author Summary

*Paramecium tetraurelia* provides an excellent model for studying the mechanisms involved in the production of non-coding transcripts and their mode of action. Different types of non-coding RNAs (ncRNAs) were shown to be implicated in the programmed DNA elimination process that occurs in this organism. At each sexual cycle, during development of the somatic nucleus from the germline nucleus, the genome is massively rearranged through the reproducible elimination of germline-specific sequences including thousands of short, single copy, non-coding Internal Eliminated Sequences (IES). Here, we demonstrate, using RNA interference, that the *TFIIS4* gene encoding a development-specific homolog of RNA polymerase II elongation factor TFIIS, is indispensable for ncRNA synthesis in the new somatic nucleus. TFIIS4 depletion impairs the assembly of a functional somatic genome and affects excision of a large fraction of IESs, which leads to strong lethality in the sexual progeny. We propose that TFIIS4-dependent ncRNAs provide an important component of the molecular machinery that is responsible for developmental genome remodeling in *Paramecium*.

## Introduction

Recent progress in high-throughput transcriptome analysis has led to a constantly growing catalog of non-coding transcripts (ncRNAs, for review see [1,2]). The prevalence of a variety of short (~20–35 nt; sRNAs) and long ncRNAs (>~200 nt; lncRNAs) has been reported in numerous organisms. However, ncRNAs differ not only in their size but also in their genomic context, cellular function and, finally, biosynthesis pathway. Large intervening/intergenic ncRNAs (lincRNAs) can be transcribed as distinct transcription units. Genomic regulatory elements also give rise to ncRNAs: transcription start site-associated short RNAs (TSSa-RNAs) or promoter upstream transcripts (PROMPTs) are produced from promoter regions, and eRNAs from enhancer regions. Regulatory short micro-RNAs (miRNAs) or longer ncRNAs can be transcribed from gene introns. Endogenous ncRNA molecules, at least partially complementary to known protein-coding transcripts, were also discovered and named natural antisense transcripts (NATs). Long ncRNAs may as well be produced from transcriptionally active pseudogenes, and can in turn yield endogenous short interfering RNAs (siRNAs) or miRNAs. Moreover, piwi-interacting RNAs (piRNAs), in *Drosophila* as in mammals, are derived from heterochromatin domains containing mostly transposable elements and degenerate transposons, and are involved in transposon silencing. To sum up, virtually all kinds of genomic regions are reported to have some transcriptional activity. For instance, around 80% of the human genome was shown to display transcriptional activity, while only a few percent consist of annotated coding regions [3]. The function of numerous ncRNAs, including those attributed to promiscuous transcription of non-coding genomic regions, still needs to be determined.

Most ncRNA production has been attributed to RNA polymerase II, although miRNAs are also transcribed by RNA polymerase III [4]. Moreover, the fidelity of transcriptional initiation by RNA polymerase II is postulated to be quite low *in vivo* and up to 90% of polymerase II initiation events may correspond to “transcriptional noise” [5], which makes it difficult to distinguish between background and functional RNAs. Some long ncRNAs were reported to be processed post-transcriptionally as mRNA transcripts–they are spliced (NeST, ANRIL) or polyadenylated (HOTTIP) [6]. Among the known sRNAs, endogenous siRNAs and miRNAs originate from double-stranded RNA precursors and are processed by enzymes displaying RNase III activity. One important question that needs to be answered is whether the synthesis of the precursor transcripts that give rise to different kinds of ncRNAs in eukaryotes requires a particular composition of the transcriptional machinery as reported in plants, where the specialized RNA polymerase IV synthesizes siRNAs and RNA polymerase V produces nascent RNAs that act as a scaffold to allow siRNAs to interact with chromatin [7].

*Paramecium tetraurelia* and other ciliates provide excellent models for studies of non-coding RNA synthesis pathways, since genome-wide transcription leading to different classes of ncRNA molecules has been reported in these unicellular organisms. Small ncRNAs and longer non-coding transcripts have been implicated in the epigenetic programming of developmental genome rearrangements that take place during assembly of the somatic genome from the germline genome [8]. *P*. *tetraurelia* houses its somatic genome in its macronucleus (MAC), which is responsible for gene expression. Two diploid micronuclei (MICs), transcriptionally inactive during vegetative divisions, harbor the germline genome and are used for the sexual exchange of DNA. At each sexual cycle, the maternal MAC is destroyed and a new MAC differentiates from a copy of the germline nucleus. During this process, the genome is massively endo-replicated (from 2n to 800n) and rearranged. Genome rearrangements include the imprecise elimination of repeated DNA elements (transposons, minisatellites) and the precise excision of over 45,000 short, single-copy Internal Eliminated Sequences (IESs) distributed both in gene-containing and non-coding regions [9]. IESs are removed by means of an extremely precise mechanism leading to the reconstitution of functional genes, which is crucial for the development of the functional new MAC and the progeny survival after sexual events (reviewed in [10,11]). It was shown that PiggyMac (Pgm), a potentially catalytically active domesticated *piggyBac* transposase, is indispensable for DNA rearrangements and involved in DNA cleavage at IES ends [12]. The extremities of *Paramecium* IESs carry very loosely conserved inverted repeats, each containing one invariant TA dinucleotide, and these signals are not sufficient to define sequence-specific excision sites across the genome [9].

Rearrangement patterns in *Paramecium* can be inherited between the old and the new MAC and this process involves a global comparison of the germline and somatic genomes that is thought to be mediated by different types of ncRNAs (reviewed in [8,13,14]). Development-specific 25-nt scnRNAs are synthesized in the MIC during meiosis from most, if not all, of the germline genome [15–17]. They are thought to be transferred to the maternal MAC, where they are probably compared through pairing interactions (“scanning”) with constitutively expressed protective ncRNAs representing a copy of the whole maternal somatic genome [18]. This process results in enrichment of MIC-specific molecules within the scnRNA population [17]. The selected scnRNAs would be transported to the developing MAC, where they might target elimination of homologous germline-specific sequences. An additional class of short ncRNAs (26–30 nt), named iesRNAs, was recently shown to be produced specifically from IES sequences in the developing new MAC and proposed to stimulate IES excision [17]. In the model for RNA-mediated control of DNA elimination in *P*. *tetraurelia*, scnRNAs have been proposed to induce epigenetic modifications of chromatin and imprint their homologous sequences for subsequent deletion *via* base-pairing to homologous nascent transcripts (e.g. IES-containing transcripts) in the new MAC [18,19], while iesRNAs would amplify the specific recognition of IES ends [17]. The biogenesis of scnRNAs and iesRNAs in *P*. *tetraurelia* involves specialized proteins related to the RNA interference machinery, including Dicer-like proteins Dcl2/Dcl3 for scnRNAs and Dcl5 for iesRNAs [15,17] and Piwi-like proteins, Ptiwi01 and Ptiwi09, which are thought to associate with scnRNAs [20]. The putative histone methyltransferase Ezl1 was recently demonstrated to be required for histone H3K27 and H3K9 trimethylation in the developing new MAC and for the correct excision of 70% of IESs [19]. However, recent reports showing that scnRNAs and iesRNAs are only required for excision of a small subset of IESs (less than 10%) [17,19] leave the question of IES recognition only partially answered, and make the catalog of components involved in this pathway–including proteins and RNA molecules—far from complete.

In this study, we addressed the question of which factors are involved in the production of ncRNAs in *Paramecium*. Since IESs are distributed throughout the genome, the “scanning” model requires ncRNA synthesis from the entire germline and somatic genomes, both from coding and non-coding regions. As a starting point, we postulated that non-coding transcription would involve specialized components of the RNA synthesis machinery, which would initiate transcription genome-wide, for instance by changing the promoter specificity of the RNA polymerase complex or its sensitivity to termination signals. A good candidate for such a transcriptional modulator is the TFIIS general transcription factor, which was shown in yeast to interact closely with Rpb1, the large subunit of RNA polymerase II (Pol II) [21]. Indeed, TFIIS plays a key role in unblocking the arrested polymerase that has backtracked along the DNA template, which leads to the displacement of the 3’ end of the nascent RNA from the Pol II active site. TFIIS stimulates cleavage of the nascent transcripts extruded from the active center by enhancing the intrinsic endonucleolytic activity of the polymerase, consequently allowing RNA synthesis to resume [22]. TFIIS, therefore, stimulates transcription elongation by shortening the duration of Pol II transcriptional pausing and facilitating transcription through the nucleosomal barrier [23]. In addition, TFIIS was also shown to play a role in the formation or/and stabilization of Pol II preinitiation complex [24,25]. TFIIS was also demonstrated to be a polymerase III general transcription factor in *S*. *cerevisiae* [26] and mammals [27], as it was discovered that TFIIS is associated with class III genes and with SINE elements. Here, we report the identification of six *P*. *tetraurelia* genes encoding TFIIS homologs, which could be grouped in four distinct families. A whole-genome survey of the transcriptome during autogamy, a self-fertilization process [28], led us to focus on the *TFIIS4* gene, which is not transcribed during vegetative growth and is specifically induced during autogamy. We provide evidence for a novel function of a TFIIS homolog in the regulation of developmentally programmed genome rearrangements. Using a combination of molecular analyses and high-throughput DNA sequencing, we show that TFIIS4 is indispensable for the synthesis of IES-containing non-coding transcripts in the new developing MAC. We propose that TFIIS4-dependent zygotic ncRNAs complete the model for RNA-mediated regulation of programmed genome remodeling in *Paramecium*.

## Results

### A multigenic *TFIIS* family in *Paramecium tetraurelia*

**Fig 1:**
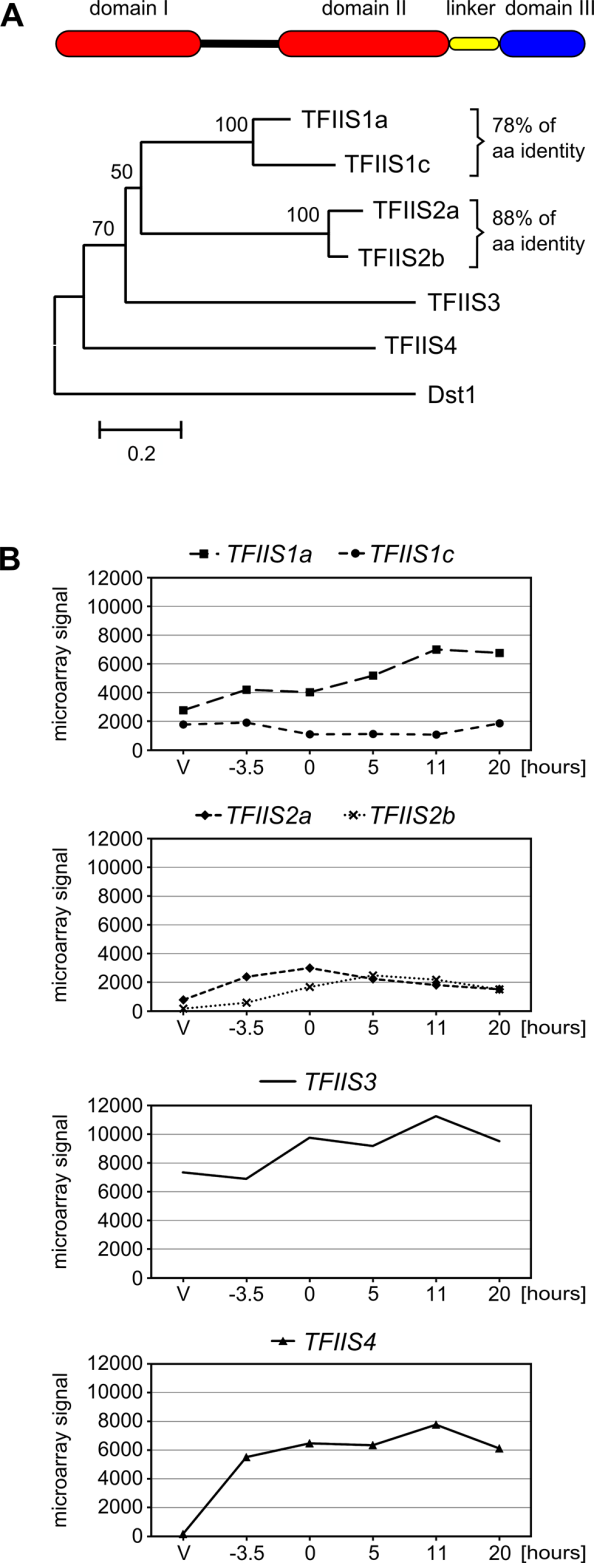
The multigenic TFIIS family in *Paramecium tetraurelia* and its expression profiles. (A) Domain organization and neighbor-joining tree of *P*. *tetraurelia* TFIIS proteins. The evolutionary history was inferred in MEGA4 [76] based on the alignment of entire protein sequences (342 positions in the final dataset) and the following parameters: deletion of gaps in pairwise sequence comparisons, uniform rates among sites, bootstrap 1000 (bootstrap values displayed next to the branches), Poisson correction. The scale is in the units of the number of amino acid substitutions per site. Accession numbers in ParameciumDB: TFIIS1a—GSPATP00003556001, TFIIS1c—GSPATP00008714001, TFIIS2a—PTETP1100023001, TFIIS2b—GSPATP00003298001, TFIIS3—GSPATP00019582001, TFIIS4—GSPATP00025792001. The Dst1 protein from *S*. *cerevisiae* was used as an outgroup. (B) Mean expression signals obtained in microarray experiment “*Paramecium tetraurelia* autogamy series 1” from [28]. Values obtained for *TFIIS2a* were recalculated using the signals obtained only for microarray probes covering the corrected gene annotation. V: vegetative; -3.5: meiosis; 0: 50% of cells with fragmented MAC; 5 to 20: 5 to 20 hours after “0” time point. Y-axis shows mean signals.

In order to find putative *Paramecium* TFIIS homologs, BLASTp and tBLASTn searches were performed against a library of predicted proteins and the *P*. *tetraurelia* somatic genome, respectively (ParameciumDB, [29]), using the sequence of Dst1p, the sole TFIIS present in *S*. *cerevisiae* [30]. We identified six genes encoding TFIIS-like proteins, representing four evolutionarily distant TFIIS families that share only 23 to 40% overall amino acid identity. Families 1 and 2 each harbor two close paralogs that were issued from ancestral whole genome duplications (WGD) of the *Paramecium* genome [31], and therefore constitute pairs of ohnologs. According to their evolutionary history, and following the Paramecium Gene Nomenclature Guidelines (see ParameciumDB), *Paramecium TFIIS* genes were named *TFIIS1a* and 1*c*, *TFIIS2a* and *2b*, *TFIIS3* and *TFIIS4* (Fig 1A).

All six *P*. *tetraurelia* proteins contain three characteristic domains (I, II and III, see Fig 1A) that can be aligned with TFIIS proteins from other eukaryotes, even though sequence identity is high only for domain III (see S1 Fig). Their predicted secondary structure seems to be conserved as in other eukaryotes–all six proteins are predicted to form a 4-helix bundle characteristic for domain I [32] with an unstructured region between domains I and II and a 3-helix bundle followed by other helixes in domain II. Domain II is tethered to domain III through a short linker region of approximately 30 amino acids predicted to form a helix. Domain III contains a predicted zinc ribbon motif which could be stabilized by a tetrad of zinc-chelating cysteine residues [21,33]. A highly conserved DE dipeptide indispensable for TFIIS protein function [34,35] is placed between two predicted beta-sheets. Based on their conserved domain organization, therefore, all *P*. *tetraurelia* TFIIS proteins seem to be active TFIIS factors.

The presence of four distant TFIIS families in *P*. *tetraurelia* is unusual compared with other species. The emergence of these four families seems to be at least as old as the speciation of the *Paramecium* genus. Indeed, all *Paramecium* species that have been sequenced so far also exhibit these four TFIIS families (S2 Fig). Four genes, one of each encoding TFIIS1, TFIIS2, TFIIS3 and TFIIS4 proteins, are present in *P*. *caudatum* [36] and *P*. *multimicronucleatum*. In species from the *P*. *aurelia complex–P*. *primaurelia*, *P*. *biaurelia*, *P*. *sexaurelia* [37], *P*. *octaurelia* and *P*. *tredecaurelia*–the exact number of WGD paralogs found in each family varies from one species to the other. *TFIIS1* is present in two or three copies; *TFIIS2* and *TFIIS3* are present in one or two copies, while in all species *TFIIS4* is encoded by a single gene (Michael Lynch’s lab data from ParameciumDB). The fact that all four TFIIS families have been conserved during *Paramecium* evolution may indicate that each family has a specific function that cannot be replaced by another family.

### Expression patterns of *TFIIS* genes reveal a strong induction of *TFIIS4* during autogamy

We investigated the expression of all six *TFIIS* genes during the sexual cycle of *P*. *tetraurelia*. Expression profiles were extracted from published microarray data [28] and showed that *TFIIS1a*, *1c* and *3* are expressed at significant levels during vegetative growth and also during autogamy (Fig 1B). In contrast, little or no expression is detected in vegetative cells for *TFIIS2a*, *2b* and *4*, while these genes are specifically induced during autogamy. All expression patterns were confirmed using northern blots for an independent autogamy time-course experiment–see S3 Fig. The different expression patterns of *TFIIS* genes make *P*. *tetraurelia* a promising model for the study of potentially divergent roles of TFIIS proteins. Interestingly, among autogamy up-regulated genes, *TFIIS4* is the most highly induced. Its transcription increases sharply early during autogamy, at the time when only vegetative and meiotic cells can be detected in the samples and remains high throughout autogamy. In all subsequent functional analyses, therefore, we paid closer attention to this gene.

### Transient specific localization of TFIIS4 in the developing MAC during autogamy

**Fig 2:**
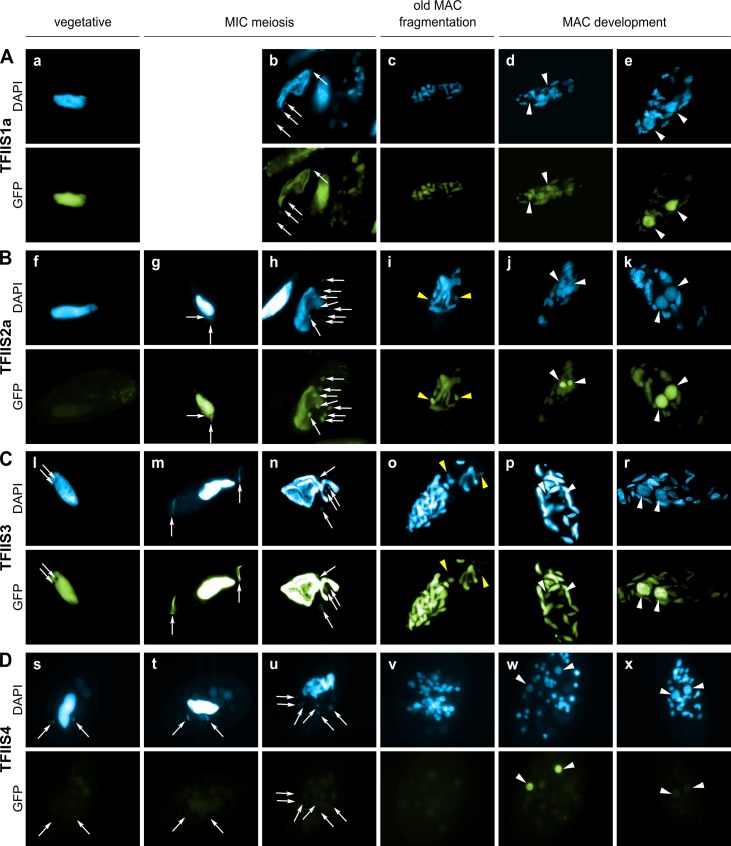
Localization of GFP fusion proteins forTFIIS1a, TFIIS2a, TFIIS3 and TFIIS4. For each transgene, representative images illustrate different developmental stages observed in a population of cells derived from a single injected caryonide. Panels a, f, l and s show vegetative cells (note that one vegetative cell is also present in the middle of panel b and on the left of panel h). All other panels show successive stages of autogamy: panels g, m and t–meiotic crescent stage; panels b and n–first meiotic division; panels h and u–cells with 8 haploid nuclei resulting from meiosis II; panels c, i, o and v–fragmentation of old MAC; panels d, j, p, and w–early MAC development; panels e, k, r and x–late MAC development. Note that panel b contains not only one meiotic cell (on the left) but also one vegetative cell (in the middle) and two cells with their fragmented old MAC (at the top and on the right). In all panels, white arrows point at MICs (some were omitted when MICs were not clearly distinguishable by DAPI staining), white arrowheads indicate new MACs. Yellow arrowheads in panels i and o point to division products of the zygotic nucleus. (A) A GFP-TFIIS1a fusion localizes to old, then new MACs. (B) A GFP-TFIIS2a fusion localizes to old MAC during meiosis, then to new MACs and is present in meiotic MICs. (C) As in B for a GFP-TFIIS3 fusion. GFP-TFIIS3 cannot be seen in division products of the zygotic nucleus. (D) A GFP-TFIIS4 fusion is essentially restricted to the new MACs specifically during early MAC development. Very weak GFP signal is visible in the old MAC during meiosis.

During sexual processes, coding and non-coding transcription take place in the three different types of nuclei that coexist in the cytoplasm of *P*. *tetraurelia*. Gene transcription progressively switches from the old MAC fragments to the developing new MACs [38], which undergo genome rearrangements. The MIC is transcribed specifically during meiosis to give rise to scnRNAs [15]. Constitutive generalized non-coding transcription takes place in the maternal MAC to produce the protective transcripts that antagonize scnRNAs [18]. During MAC development, short iesRNAs are produced from putative precursor transcripts synthesized in the new developing MAC [17]. Finally, nascent non-coding transcripts produced from the new developing MAC were proposed to serve as substrates for the pairing of MIC-restricted scnRNAs to guide DNA elimination in the developing new MACs [13,18]. In order to gain insight into the role of each TFIIS family in these different nuclear compartments, GFP fusions were constructed for each of the six proteins and expressed under the control of their respective endogenous regulatory regions. The cellular localization of GFP-TFIIS proteins was monitored by injecting each transgene into the macronucleus of vegetative cells and following the GFP fluorescence during vegetative growth and throughout autogamy (Fig 2). All proteins were shown to be nuclear, but they localized to different nuclear compartments.

TFIIS1a is present in the vegetative MAC (Fig 2A). During autogamy the GFP fluorescence progressively shifts from the fragmented old MAC to the developing new MAC (Fig 2B–2E). Localization of its close paralog TFIIS1c is similar, although it seems to disappear more abruptly from the fragmented old MAC (S4C Fig), before it eventually accumulates in the new MACs (S4D and S4E Fig). In conclusion, proteins from the TFIIS1 family shift from the old to the new MAC during autogamy. This localization pattern may reflect a possible role of TFIIS1a and 1c in mRNA synthesis and gene expression in the somatic nucleus.

Protein fusions encoded by the autogamy up-regulated genes *TFIIS2a* and *2b* accumulate during meiosis, both in the old MAC and the meiotic MICs (Fig 2G). GFP fluorescence was also detected in all meiotic products (Figs 2H, S4G and S4H, respectively). Both TFIIS2 fusions eventually concentrate in the developing new MACs (Figs 2J, 2K and S4J, S4K). TFIIS3 follows the same localization pattern with clear presence in the vegetative MAC. The TFIIS2 and TFIIS3 families are present wherever transcription takes place—in the old and new MACs and in meiotic MICs—and may be associated with coding or non-coding transcription. In particular, the presence of TFIIS2 and TFIIS3 in the MICs during meiosis suggests their possible involvement in the non-coding transcription that gives rise to scnRNAs.

**Fig 3:**
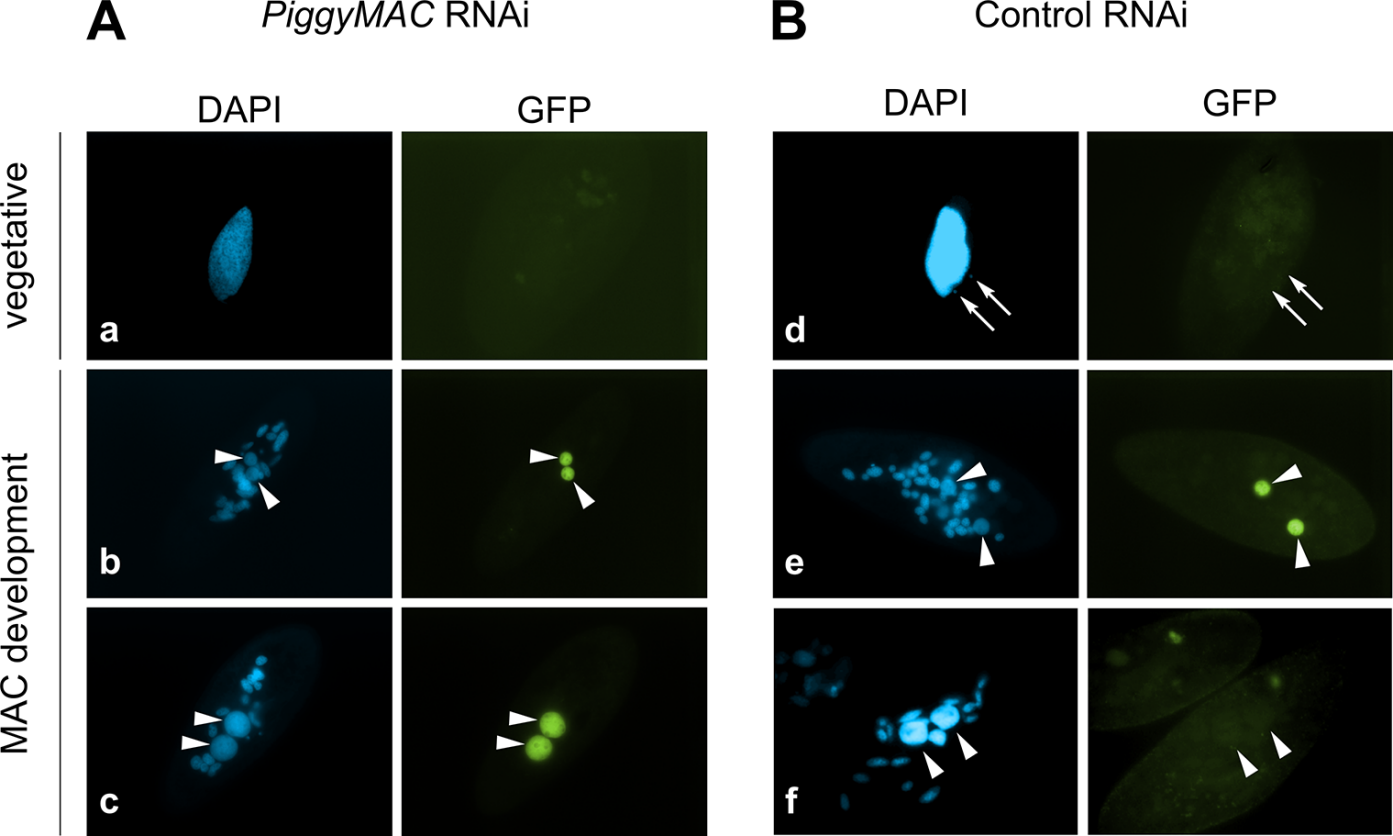
Localization of the GFP-TFIIS4 fusion protein upon *PiggyMac* RNAi. New developing macronuclei (new MAC) are indicated by white arrowheads, while white arrows point at MICs that are clearly visible only in panel d. Panel a and d show vegetative cells, b and e: early MAC development, c and f: late MAC development. (A) Cells silenced for *PiggyMac*. The efficiency of *PiggyMac* silencing was confirmed by the observation of 100% lethality in the sexual progeny. (B) Control experiment, in which the nonessential *ICL7* gene was silenced. The silencing of *ICL7* gene does not interfere with autogamy (see Table 1) and does not influence the localization of TFIIS4 relative to cells grown in standard *K*. *pneumoniae* medium.

Consistent with the expression pattern of *TFIIS4*, no fluorescence was observed in vegetative cells injected with a *GFP-TFIIS4* fusion transgene (Fig 2Ds). During autogamy, trace amounts of the protein were detected in the old MAC during MIC meiosis (Fig 2T), then GFP fluorescence accumulated in the new MACs at early stages of MAC development (panel w), and diminished at later stages (panel x). No staining of the MICs was detected at the crescent stage and after meiotic divisions (panel t and u). This very peculiar localization may reflect a specific transcription-related function connected with the DNA elimination process that starts by the time TFIIS4 appears in the new MAC. In this respect, we noted that the presence of GFP-TFIIS4 in the developing MAC is very transient, since it disappears at late stages, when genome rearrangements are probably completed. Moreover, no lethality was observed in the post-autogamous progeny of injected cells (survival rate of six independently injected clones similar to non-injected controls), indicating that the GFP-TFIIS4 fusion did not interfere with normal progression of the sexual cycle. To get further insight into the timing of TFIIS4 localization relative to genome rearrangements, we repeated the experiment in PiggyMac-depleted cells, in which DNA elimination is inhibited [12]. Under these conditions, GFP-TFIIS4 persisted in the new MACs until the latest stages of autogamy (Fig 3). Taken together, these observations suggest that GFP-TFIIS4 accumulates in the new MACs before IES excision, and disappears once IESs have been removed.

### Expression of *TFIIS4* is essential for the successful completion of autogamy

*Paramecium* TFIIS factors exhibit different expression and localization patterns during vegetative growth and sexual processes. To check if any of them have an essential function during autogamy, we silenced each *TFIIS* gene, by feeding *Paramecium* cultures on dsRNA-overproducing bacteria to trigger RNA interference [39]. For *TFIIS2a* and *2b*, we also performed double silencing experiments by mixing induced bacteria designed to silence individual genes. The effect of each RNAi was first examined for ~8 vegetative divisions by monitoring cell division rate and general morphology, as described in [40]. None of the silencing experiments gave an obvious phenotype during this period of vegetative growth. In a second step, autogamy was induced by starvation, and the survival of sexual progeny was checked following transfer of individual autogamous cells to standard medium (Table 1). Inactivation of individual genes from families 1, 2 or 3 did not produce any visible phenotype. For autogamy up-regulated *TFIIS2a* and *2b* genes, we did not observe any phenotype when both genes were silenced together. It may indicate that TFIIS2 proteins are not essential or display functional redundancy with other TFIISs, for example TFIIS3, which shows a similar localization pattern. It is of course possible that some *TFIIS* genes were not completely silenced by the RNAi method used in our study and, therefore, that no phenotype was revealed in our screen. We should also note that, in other model systems, a mutation of *TFIIS* very often does not give strong phenotypes: in the yeasts *S*. *cerevisiae* and *S*. *pombe*, null mutants in the single copy *TFIIS* gene are viable under standard laboratory conditions, but sensitive to the nucleoside analog 6-azauracil [41,30]. In contrast, single RNAi against *Paramecium TFIIS4* led to strong lethality in post-autogamous progeny, with only 15% normally growing survivors. Most of the remaining surviving progeny was sick, grew slowly, failed to divide normally, and finally died after a few divisions. We conclude that *TFIIS4* shows a clear-cut RNAi phenotype during autogamy and a specific localization of its encoded protein in the developing new MAC. Cytological observation of DAPI-stained cells confirmed that TFIIS4 depletion does not impair the differentiation of new MACs, which are formed and amplify their DNA normally (S5 Fig).

**Table 1:** RNAi-screening for essential *TFIIS* genes.

Targeted gene		*TFIIS1a*	*TFIIS1c*	*TFIIS2a*	*TFIIS2b*	*TFIIS2a* and *TFIIS2b*	*TFIIS3*	*TFIIS4*	*ND7*	*ICL7*	none
Progeny	% wild type	94	91	94	86	89	90	15	97	97	87
	% sick	1	0	1	2	3	2	10	0	0	2
	% death	5	9	5	12	8	8	75	3	3	11
Total cells		192	192	192	192	192	192	493	282	210	222
Number of experiments		3	3	3	3	3	3	13	6	7	4

Survival test of post-autogamous cells submitted to RNAi against all *TFIIS* genes and control non-essential genes—*ICL7* and *ND7*. The last column “none” corresponds to the control grown in standard non-feeding *K*. *pneumoniae* medium. For each condition, the number of replicate experiments is indicated in the last line. In one replicate experiment, wild-type survivors were systematically tested for MAC regeneration (as previously described [52]) and all turned out to be true postautogamous cells.

### TFIIS4 is required for excision of a subset of IESs

**Fig 4:**
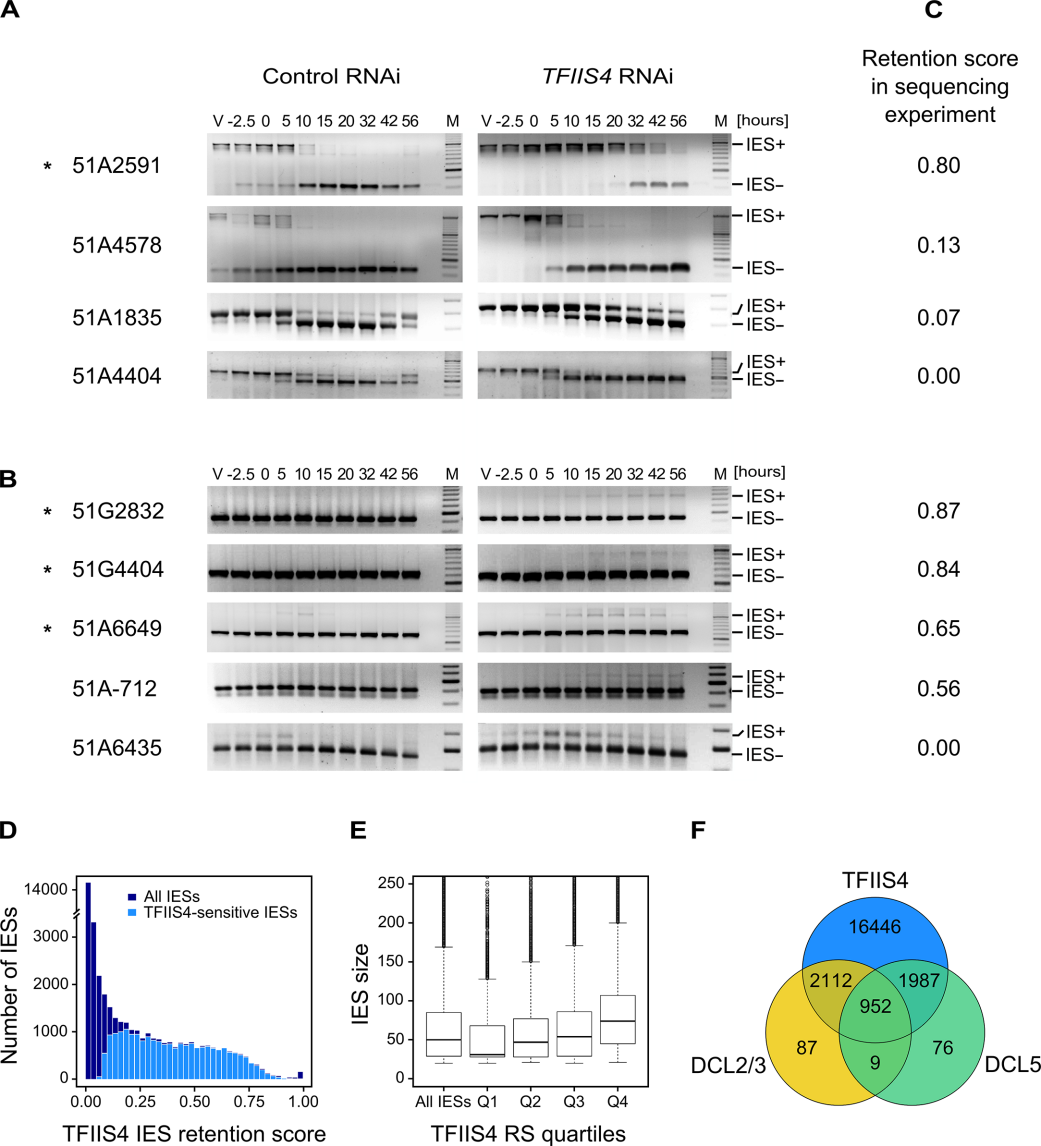
Analysis of IES excision in *TFIIS4*-silenced cells. (A) PCR analysis of the excision of IESs located in the surface antigen gene *A* ^*51*^ using primers located around each IES. In each panel, the larger fragment corresponds to the non-excised form (IES+), the smaller fragment to the excised form (IES-). Known maternally controlled IESs are labeled with an asterisk. The autogamy time-course experiment was performed using a strain harboring a somatic (macronuclear) deletion of part of surface antigen gene *A* ^*51*^, which overlaps 3 tested IESs – 51A1835, 51A4404, 51A2591 and partially 51A4578. In this experiment, we obtained 93% lethality in post-autogamous progeny of *TFIIS4*-silenced cells. (B) As in A for IESs located in other regions. The PCR products corresponding to each IES- form are amplified mostly from the fragments of the old MAC. Oligonucleotide sequences are listed in S2 Table. (C) IES retention scores calculated from the genome-wide sequencing of DNA extracted from purified nuclei of cells silenced for *TFIIS4* during an independent RNAi experiment (87% lethality in post-autogamous progeny). (D) Superimposed histogram of TFIIS4 retention scores for all IESs (dark blue) and for IESs that are significantly retained in TFIIS4-depleted cells (light blue). Around 25,000 IESs are not significantly affected by the inactivation of *TFIIS4* and a large fraction of IESs exhibits a retention score equal to 0. For TFIIS4-dependent IESs, retention scores are almost uniformly distributed between 0.1 and 0.7. (E) The graph shows a positive correlation between IES size and retention score in *TFIIS4* RNAi. The box plot displays the IES size distribution for all IESs and for each of *TFIIS4* retention score (RS) quartiles. The median retention score (horizontal line inside the box) and the first (top of box) and third (bottom of box) quartiles are shown. Range of RS for particular quartiles are as follows: Q1: [0–0.01[; Q2: [0.01–0.12[; Q3: [0.12–0.39[; Q4: [0.39–1.00]. The medians are significantly different between all the groups (p < 2e-40). (F) Venn diagram of significantly retained IESs after *TFIIS4*, *DCL5* or *DCL2/3* silencing. Almost all IESs that are dependent upon Dcl2/3 or Dcl5 for their excision are also dependent upon TFIIS4.

**Fig 5:**
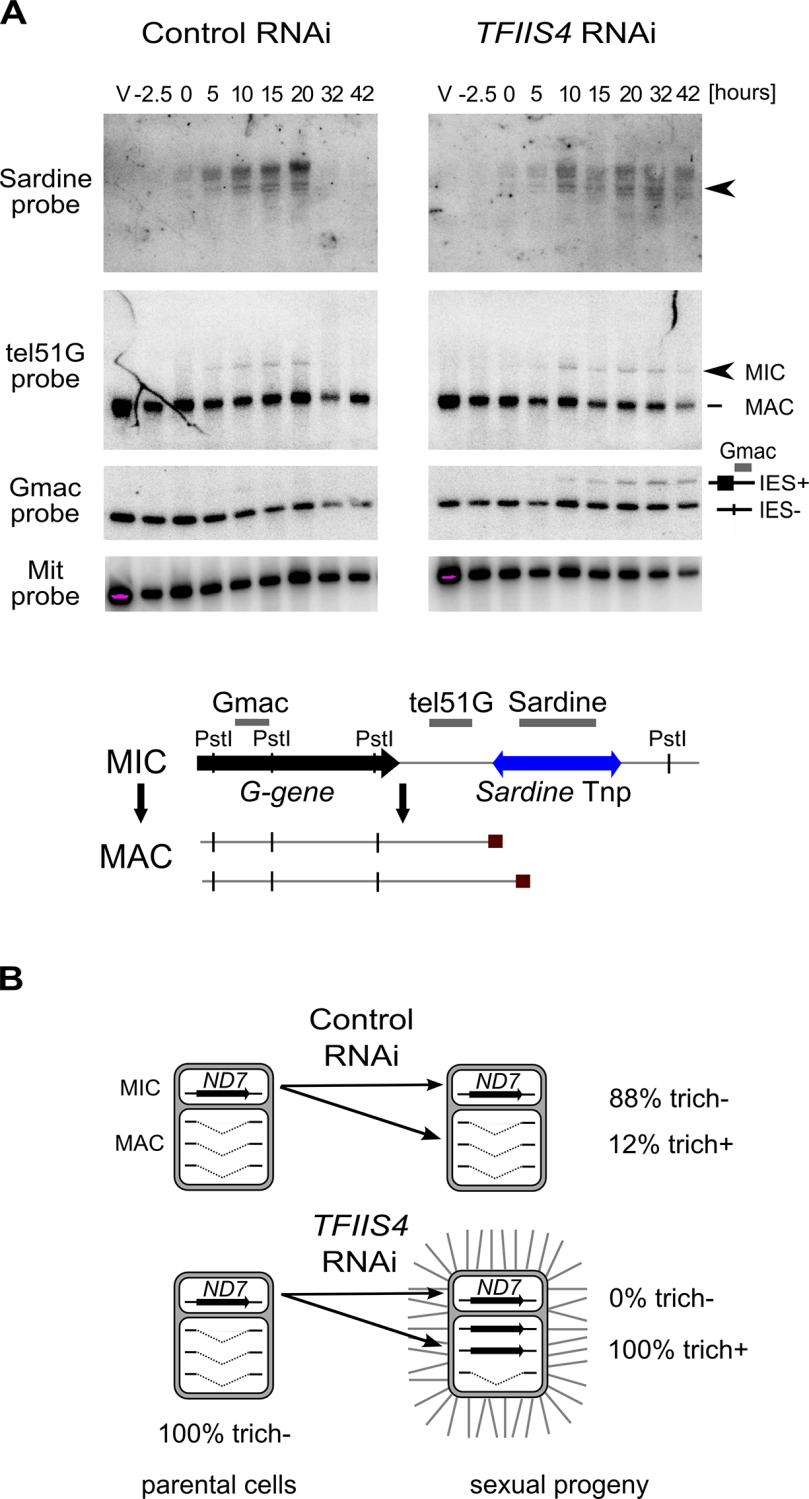
Inhibition of imprecise genome rearrangements in *TFIIS4*-silenced cells. (A) Southern blot analysis of *Pst*I-restricted genomic DNA from the autogamy time-course experiment in which 51mt8 *∆A∆ND7* cells were silenced for *ICL7* and *TFIIS4*. Autogamy stages are marked as follows (see S6 Fig): V–vegetative cells, -2.5 –cells during meiosis, 0 to 56 –autogamy stages in hours (with T0 corresponding to the stage when around 50% of cells harbor a fragmented old MAC). The blot was hybridized with probes corresponding to different sequences from the locus carrying the *G* surface antigen gene: Sardine, reveals the *Sardine* transposon located downstream of the *G*-gene (arrowhead) and other related transposon sequences in the genome; tel51G hybridizes to the non-fragmented germline chromosome (upper band) and its fragmented MAC version obtained by telomere addition downstream of the *G* gene (lower band); Gmac hybridizes with fragments containing (upper band) or not (lower band) IES 51G4404. The same blot was hybridized with a mitochondrial DNA probe (Mit probe) as a loading control. (B) Phenotypic test of the ability to discharge trichocysts in the sexual progeny of cells carrying a macronuclear deletion of the *ND7* gene, following *ICL7* or *TFIIS4* gene silencing. *TFIIS4* silencing restores a wild-type trich+ phenotype, most probably due to lack of inheritance of the macronuclear deletion.

Because half of the genes in *P*. *tetraurelia* are interrupted by at least one IES [9], the development of a functional new MAC depends upon the completion of IES excision. We therefore tested the excision of several known IESs by PCR, using primers located in the flanking MAC sequences upstream and downstream of each particular IES. In this experiment, we used a strain carrying a somatic deletion of part of surface antigen gene *A*, in which a region containing three IESs is absent from the maternal MAC [42]. We extracted genomic DNA and total RNA samples during an autogamy time-course of this strain silenced either for a non-essential control gene or for *TFIIS4* (S6A Fig). The efficiency of *TFIIS4* silencing was confirmed by northern blot hybridization of total RNA (S6B Fig), and genomic DNA was used to monitor genome rearrangements at the molecular level. In the control experiment, the use of a ∆*A* strain allowed us to detect *de novo* IES excision junctions for this locus (Fig 4A). In the *TFIIS4* RNAi, we observed a strong delay in excision of IES 51A2591 and very low amounts of excision products (IES-) relative to the control RNAi. Excision was also delayed to some extent for IESs 51A4578 and 51A1835, whereas we observed a normal elimination profile for 51A4404. Other IESs located outside the region of the ∆*A* macronuclear deletion were also tested (Fig 4B). For these IESs, due to the presence of rearranged DNA in the old MAC, we could only monitor IES retention during autogamy. Based on the persistence of the IES+ form at late time-points, excision of IESs 51G2832, 51G4404, 51A6649 and 51A-712 was found to be inhibited in *TFIIS4*-silenced cells, while another IES (51A6435) seemed to be eliminated normally. Interestingly, all the known maternally controlled IESs that we tested [43] are affected by silencing of the *TFIIS4* gene (indicated by an * in Fig 4A and 4B). In particular, we confirmed the retention of IES 51G4404 by Southern blot hybridization (Fig 5A). In conclusion, our molecular results indicate that excision of some IESs is inhibited by *TFIIS4*-silencing to various extents, while other IESs are eliminated normally.

To get a quantitative and genome-wide insight into the dependence of IES excision upon TFIIS4, we performed high-throughput sequencing of DNA extracted from a nuclear preparation enriched for new MACs of *TFIIS4*-silenced autogamous cells (obtained from an independent RNAi experiment), as described in [9]. As a control we used sequencing data for the DNA sample obtained from autogamous cells of the same strain, but with no silencing ([19], see Materials & Methods for details). In both datasets, IES retention scores were determined for each IES by calculating the ratio of IES-containing reads (IES+) over the sum of: (i) the number of reads that map to the IES excision junction (IES- reads), and (ii) the number of IES-containing reads (IES+). Hence a retention score of 0 means complete excision whereas a score of 1 means complete retention of the IES as described in [19]. The results are in good agreement with the above molecular data: IESs shown to be retained by PCR or Southern blot hybridization exhibit higher retention scores in the sequencing experiment (Fig 4C). To ensure that the IES retention observed in the TFIIS4 RNAi sample is indeed due to the silencing of this gene, we performed statistical comparison of IES retention scores between control condition and TFIIS4-RNAi (for details see Materials and methods). This statistical analysis revealed that ~21,500 IESs (48%) are sensitive to *TFIIS4* silencing, with a very wide distribution of IES retention scores (Fig 4D). Among the set of TFIIS4-dependent IESs, we found all five maternally controlled IESs that were identified in previous experiments [43] and all IESs that were shown to be dependent upon the presence of the WG/GW-repeat protein Nowa1/2 [17,44]. Moreover, almost all TFIIS4-sensitive IESs (96%) can be found in a larger set of IESs dependent on the putative histone methyltransferase Ezl1 [19] (Table 2). TFIIS4-dependent IESs are characterized by higher average retention scores in *EZL1*-silenced cells relative to IESs that do not depend upon TFIIS4. However, we did not observe a strong correlation of IES retention scores between *TFIIS4* and *EZL1* silencing experiments (S7A Fig). addition, we noticed a correlation between IES size and IES sensitivity to *TFIIS4* silencing: IESs with higher retention scores (i.e. strongly dependent upon TFIIS4) tend to be longer (Fig 4E). However, because of a wide distribution of the TFIIS4 retention scores for all IES size groups (S7B Fig), the parallel increase of the retention score with increasing IES size is not as obvious as reported for *EZL1* RNAi [19]. The apparent overlap between the requirements for TFIIS4, Nowa1/2 and Ezl1 for IES excision suggests that TFIIS4 may be implicated in the control of IES excision *via* the same RNA-related pathway as the one in which nucleosomes are marked by methylation.

**Table 2:** Global analysis of genome rearrangements in *TFIIS4* silencing—comparison with *EZL1*, *DCL2/3* and *DCL5*.

	All IESs	TFIIS4-sensitive IESs	EZL1-sensitive IESs	DCL2/3-sensitive IESs	DCL5-sensitive IESs
Number of IESs	44928	21497	31481	3160	3024
Average IES length [nt]	79.1	93.5	92.3	200.8	57.7
Number of TFIIS4-sensitive IESs	21497	21497	20705	3064	2939
Percentage of TFIIS4 sensitive IESs among the category	48%	100%	66%	97%	97%
Average TFIIS4 retention score	0.22	0.40	0.29	0.61	0.55

IESs retained in different samples (in column) are described according to their size and their presence in the TFIIS4-dependent IES set. Almost all IESs retained in other RNAi experiments are also retained in *TFIIS4* silencing, with high retention score. Apart from Dcl5-dependent IESs, IESs retained following *TFIIS4* RNAi are larger (on average) than the overall set.

### Involvement of TFIIS4 in imprecise DNA elimination

In addition to the precise excision of single-copy IESs, genome rearrangements include the elimination of repeated DNA sequences such as transposable elements. Two families of *Tc1*/*mariner*-related transposons were identified in the part of MIC-specific sequences that are removed imprecisely during MAC development: *Sardine* and *Thon* [9]. We first used Southern blot hybridization with a specific probe to monitor the transient amplification and the elimination of *Sardine* transposons from the developing MAC during autogamy (Fig 5A). In *TFIIS4*-silenced cells, we observed an accumulation of the signal corresponding to *Sardine* transposons, indicating at least a partial block of transposon elimination. One copy of the *Sardine* is located downstream of a telomere addition site and its elimination is associated with chromosome fragmentation. Using a macronuclear subtelomeric probe (tel51G, see [12]), we confirmed that retention of this copy of the *Sardine* upon *TFIIS4* silencing correlates with the persistence of non-fragmented forms of the chromosome (Fig 5A). We also used our genome-wide sequencing data to estimate the fraction of repeated sequences that require TFIIS4 for elimination. Since the germline reference genome is not available for *P*. *tetraurelia*, we used an unrearranged version of the genome, previously assembled from the sequencing experiment following PGM depletion, as our reference [9]. We mapped the sequencing reads from the control sequencing (wild type genome) as well as PGM- and TFIIS4-knockdowns on this unrearranged reference genome and measured the complexity of the regions present in the PGM and TFIIS4 samples but not in the control sample (see legend of S3 Table for the entire procedure). We found that 64% of the MIC-restricted sequences need TFIIS4 for their elimination (S3 Table). We conclude, therefore, that TFIIS4 is necessary for removal of some, but not all repetitive sequences. A true micronuclear assembly along with its annotation would be required for further analysis of the role of TFIIS4 in imprecise DNA elimination during MAC development.

Imprecise DNA elimination is also involved in the maternal inheritance of somatic deletions, as was demonstrated for the macronuclear deletion of the *ND7* gene [45]. The *ND7* gene encodes a trichocyst discharge protein that is non-essential during autogamy. Its micronuclear version harbors one TFIIS4-independent IES. We tested the inheritance of a macronuclear *ND7* deletion during autogamy of *TFIIS4*-silenced cells. In a control RNAi we observed that 88% of sexual progeny (35 cells out of 40) retained the mutant phenotype, which is detectable only when all copies of the *ND7* gene are deleted from the new MAC (Fig 5B). Following *TFIIS4* silencing, all post-autogamous progeny (30 cells out of 30) switched back to a wild-type phenotype, indicating that inheritance of the ∆*ND7* macronuclear deletion is strongly inhibited. Although it does not allow precise quantification, this experiment indicates that TFIIS4 is involved in the maternal inheritance of imprecise somatic deletions.

### TFIIS4 is involved in IES transcription in the new MAC

According to the localization of a GFP fusion, TFIIS4 appears in the new MAC at an early developmental stage, which possibly coincides with the activation of global transcription in the new MAC and may precede the start of IES excision [38,46]. Given these observations, we considered two possibilities: TFIIS4 is required for the synthesis of non-coding IES transcripts prior to IES excision, or for the start of coding transcription in the new MAC.

We first examined whether TFIIS4 plays any role in the production of IES transcripts in the developing new MAC. Indeed, at early stages of MAC development, IES sequences are still present in the yet non-rearranged genomic DNA and a large fraction of genes, therefore, cannot produce functional mRNAs. IES transcripts were detected as soon as the new MACs were observed for one particular IES, 51G4404 [18], suggesting that they likely originate from the developing new MAC before IES excision. However, because cells at different autogamy stages coexist at each time-point, the exact origin of IES transcripts–and their putative cellular function—has remained unclear. We hypothesized that, during this period of time, non-coding IES-containing (IES+) transcripts may be produced in a TFIIS4-dependent manner. To test this hypothesis, we performed RT-PCR experiments for three IESs: the maternally controlled IESs 51G4404 and 51A6649, which belong to the set of TFIIS4-dependent IESs, and the non-maternally controlled IES 51A4404, which does not depend upon TFIIS4 for excision. We used total RNA samples isolated during the autogamy time-course experiments described above (Figs 4, 5 and S6), in which *TFIIS4* or a control gene were silenced.

**Fig 6:**
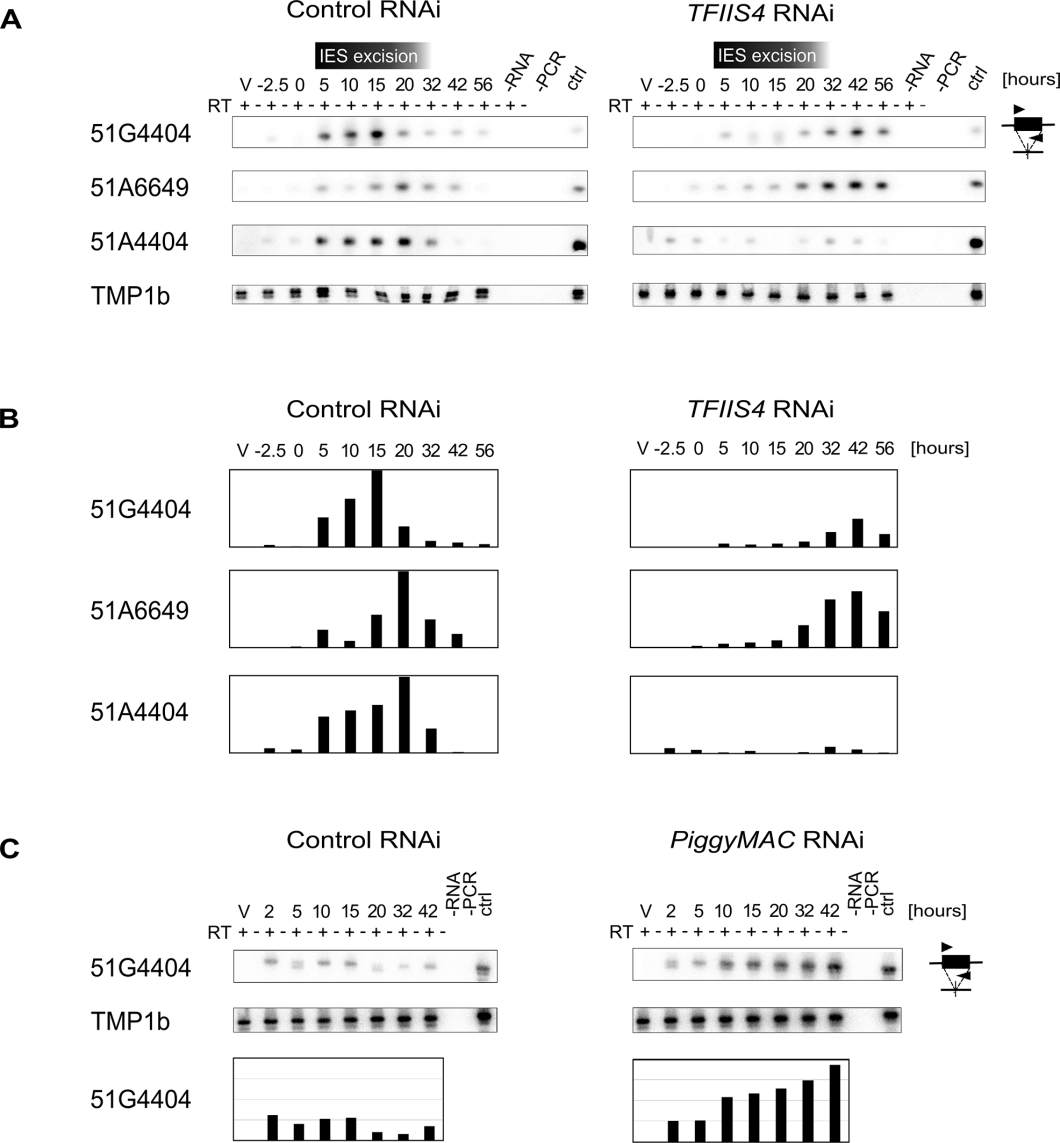
Detection of IES-containing (IES+) transcripts. (A) RT-PCR and Southern blot detection of IES-containing transcripts (IES+) in a control culture (cells silenced for *ICL7* gene expression) and in *TFIIS4*-silenced cells. Autogamy stages are marked as in S6 Fig: V–vegetative cells, -2.5 –cells during meiosis, 0 to 56 –autogamy stages in hours. Time-window when IES excision take place based on PCR shown in Fig 4 is indicated. PCR primers were located within each tested IESs: 51G4404, 51A6649 and 51A4404. The TMP1b panel shows the RT-PCR signal obtained for the constitutively expressed gene encoding trichocyst matrix protein TMP1b. (B) Histograms showing the normalization of IES+ signals shown in (A) with TMP1b mRNA. (C) Detection of IES-containing transcripts (IES+) with PCR primers located within IES 51G4404 in a control experiment, in which the *ND7* gene was silenced, and in *PiggyMac*-silenced cells. See S8 Fig, panel B for details about autogamy stages.

In the control RNAi, IES+ transcripts were detected for all three IESs starting from T5 until T20-T32 (Fig 6A and 6B), which coincides with the early stages of MAC development, when IES excision takes place (see Fig 4A). Upon *TFIIS4* silencing, practically no transcripts were detected before T20 during autogamy and only very delayed transcription was observed for IESs 51G4404 and 51A6649 starting from T32 until T56 (Fig 6A and 6B). For IES 51A4404, transcripts were hardly detectable at any autogamy time-point. We conclude, therefore, that the synthesis of IES+ transcripts during IES excision is strongly repressed in TFIIS4-depleted cells for all tested IESs. Interestingly, excision of IESs 51G4404 and 51A6649 is strongly inhibited by *TFIIS4*-silencing (see Fig 4B) and, as a consequence, these IESs are amplified together with MAC-destined DNA during autogamy. The detection of higher amounts of their corresponding transcripts at late time-points may result from the retention of these IESs in the genome of the new MAC, when somatic mRNA transcription eventually starts in this nucleus. Alternatively, it may also be explained by the fact that RNAi-mediated silencing of *TFIIS4* becomes weaker at late autogamy (as confirmed by northern blots, see S6B Fig). In contrast, IES 51A4404 is excised normally in TFIIS4-depleted cells and, therefore, cannot be transcribed during late autogamy due to the lack of a transcription substrate. These results point towards the possibility that probably all IESs—maternally or non-maternally controlled, located in different regions of the genome—are transcribed during genome rearrangements in a TFIIS4-dependent manner. However, all IESs do not require the presence of TFIIS4 to be excised. Based on the study of a GFP-TFIIS4 fusion, TFIIS4 shows a specific, but transient, localization in the new MAC. IES+ transcripts are detected as a peak during macronuclear development. This similar timing and the specific localization of TFIIS4 strongly suggest that the IES+ transcripts that are detected in our RT-PCR experiments mostly originate from the new MAC. To verify this hypothesis, we monitored IES transcription in cells silenced for *PiggyMac* expression, in which all IESs are retained in the developing MAC. Consistent with the persistence of a GFP-TFIIS4 fusion in the new MACs of *PGM*-silenced cells until late stages of autogamy (Fig 3), we observed an accumulation of IES+ transcripts relative to a control silencing (Fig 6C). Taken together these data indicate that TFIIS4-dependent IES transcripts are produced in the new developing MAC before IES excision.

### No significant role of TFIIS4 in the synthesis of other developmental transcripts

**Fig 7:**
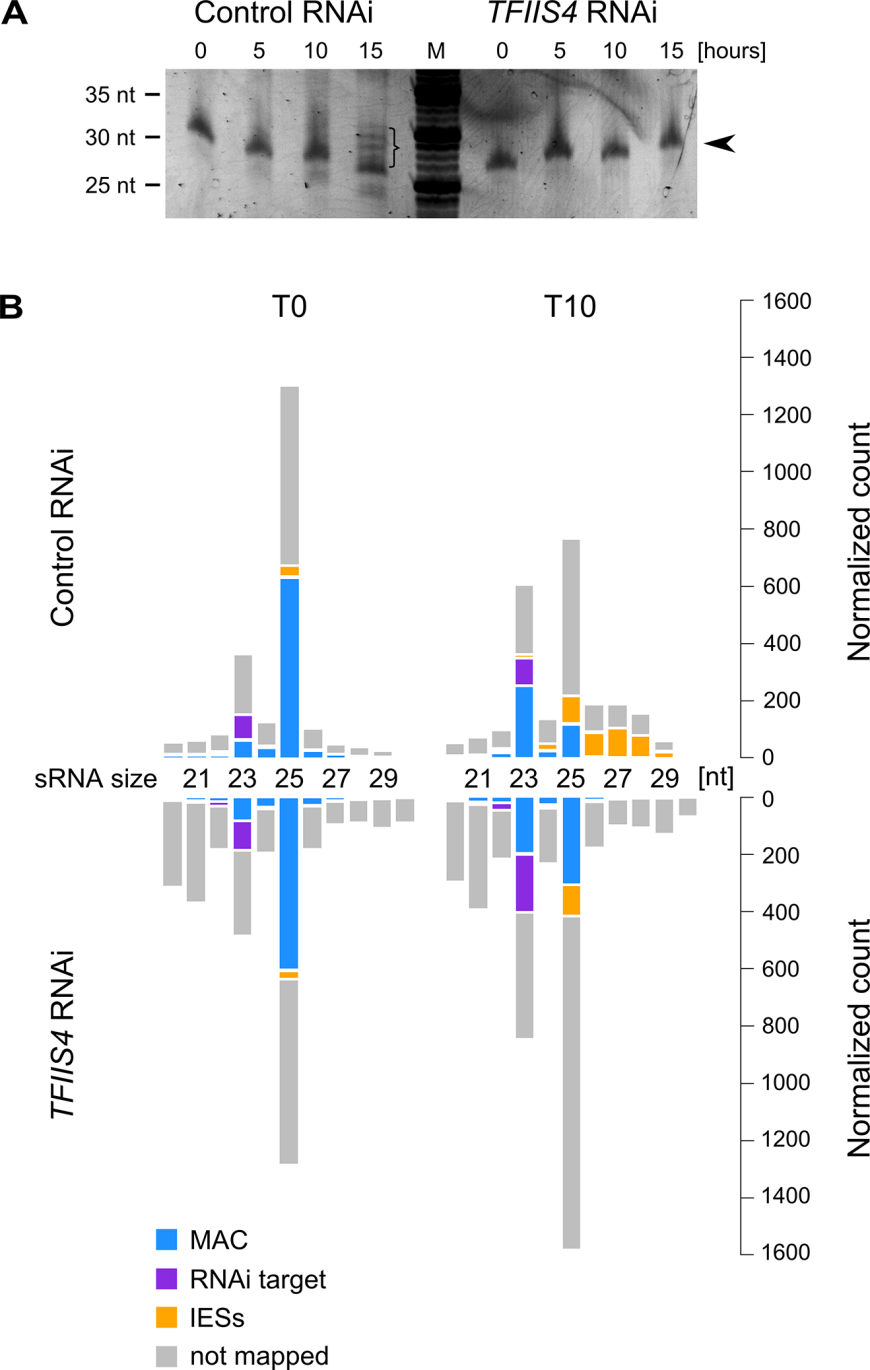
Analysis of sRNA populations in *TFIIS4*-silenced cells. (A) Total RNA samples corresponding to the T0, T5, T10 and T15 time-points from the above experiment were run on a denaturing 15% polyacrylamide-urea gel. After electrophoresis the gel was stained with SYBR Gold (Invitrogen). M: DNA Low Molecular Weight Marker (USB). Arrowhead points to the ~25 nt signal that was shown to correspond to the fraction of scnRNAs [15]. In the control, at the T15 time-point, additional bands corresponding to 26–30 nt iesRNAs are present (indicated by a bracket). In *TFIIS4*-silenced samples iesRNAs can clearly not be seen. (B) Small RNA libraries corresponding to the T0 and T10 time-points from the above experiment were sequenced and mapped to the reference genomes (*P*. *tetraurelia* MAC reference genome and MAC+IES reference genome). The top panel corresponds to a control culture (cells silenced for *ICL7* gene expression), while results for *TFIIS4*-silencing are shown below. Histograms show normalized number of sRNA reads that match to: the target silencing regions (*ICL7* or *TFIIS4* gene, respectively) – in purple; the rest of MAC genome – in blue; all annotated IESs – in yellow; all other not mapped sRNA – in gray.

Two other types of regulatory ncRNAs were previously reported to participate in the control of genome rearrangements: protective maternal MAC transcripts, which are an RNA copy of the rearranged somatic genome, and the deletion-inducing scnRNAs, which are produced from the non-rearranged germline genome during MIC meiosis [15,18]. We were able to exclude any role of TFIIS4 in the biosynthesis of either type of ncRNA. Indeed, similar levels of constitutive maternal MAC transcripts were detected by RT-PCR in a control RNAi and in a *TFIIS4* RNAi (S8A Fig). We did not detect any difference either in the global amounts of 25-nt scnRNAs between the two conditions, as revealed by SYBR Gold-staining of polyacrylamide gels (Fig 7A). These data are fully consistent with the absence of TFIIS4 from the MICs, and with a role of TFIIS4 downstream the synthesis of maternal MAC transcripts and scnRNAs. As *TFIIS4* seems to be expressed before new MACs are formed and may be present in low amounts in the maternal MAC, we decided to test the possibility that TFIIS4 is involved in scnRNA selection, which takes place in this compartment and results in enrichment of the scnRNA population in germline-specific sequences. We therefore used high-throughput RNA sequencing to compare sRNA populations present in the cell in early autogamy (T0) and at later time-point (T10) in the time-course experiment described above (Fig 7B) and in a biological replicate (S8C Fig). All sRNA reads obtained for *TFIIS4* silencing and a control RNAi were mapped to reference genomes: MAC and MAC+IES. Eventually, read counts mapping to the MAC or IES were normalized to the total number of reads mapping to the genome (MAC or IES). Our results clearly show that 25-nt scnRNAs are produced normally upon TFIIS4 depletion, since the number of reads matching the genome is similar between *TFIIS4*-RNAi and the control silencing. Moreover, we observed that scnRNAs became enriched in germline-specific sequences under both conditions, indicating that the scanning process takes place as previously published [17], even in TFIIS4-depleted cells. The increase in the relative IES/MAC ratio of scnRNAs between early and later time-point (T and T10) was higher than 7-fold for the control and higher than 5-fold in the *TFIIS4*-RNAi (see Figs 7B and S8C). We conclude, therefore, that TFIIS4 is neither involved in scnRNA production in meiotic MICs, nor in the scnRNA selection that is thought to take place in the maternal MAC. In contrast, we noticed that the recently described iesRNAs [17], which are clearly visualized at the T15 time point in the control RNAi (Fig 7A), are practically absent in *TFIIS4*-silenced cells. In agreement with this observation, we obtained a significant number of 26–30 nt reads that mapped to IESs at T10 time-point in the control RNAi, while in *TFIIS4*-silencing these sequences were clearly missing (Figs 7B and S8C) One explanation may be that TFIIS4-dependent IES transcription in the new MAC provides precursors for Dcl5-dependent iesRNA synthesis. Alternatively, iesRNAs may be produced from excised IESs and their production would be inhibited due to a block in excision of TFIIS4-dependent IESs. It is important to note that the disappearance of iesRNAs cannot be the sole reason for defective IES excision upon *TFIIS4*-silencing, since we observe much stronger phenotype in *TFIIS4*-RNAi than in *DCL5*-RNAi–both in the IES retention and the cell lethality.

Finally, we investigated the possible role of TFIIS4 in the transcription of protein-coding genes known to be involved in IES excision and focused on *NOWA1/2* and *PiggyMac*. Previous studies using GFP fusion transgenes introduced in the old MAC indicated that expression of *NOWA1* and *PGM* originates, at least in part, from the old MAC [12,44]. Therefore, we did not expect that depletion of TFIIS4, which localizes preferentially in the new developing MAC during autogamy, could lead to a strong effect on *NOWA1/2* and *PGM* expression. Indeed, we observed by northern blot hybridization that a *TFIIS4* RNAi does not cause any dramatic change in the level of *NOWA1/2* mRNA relative to a control RNAi (S9A Fig). We obtained the same result for *PiggyMac* mRNA (S9A Fig). At the protein level, we confirmed that a Pgm-GFP fusion is detected similarly to the control in TFIIS4-depleted cells (S10 Fig). Taken together, there is no reason to believe that the defect in IES excision observed in *TFIIS4* RNAi is due to depletion in Nowa1/2 or PiggyMac. The role of TFIIS4 in coding transcription was studied at the genome-wide level by performing a single microarray hybridization experiment using RNA samples extracted during vegetative growth and at five time-points during the autogamy time-course shown in S6 Fig. We focused on the ~5000 genes showing the most significant changes in their expression during autogamy under standard conditions [28]. We did not notice important global changes in the variations of mean transcript levels between the control and *TFIIS4* RNAi experiments (S9B Fig), especially for the early activated (maximum induction at the T-2.5 and T0 time-points), the late autogamy genes (induced at T10 and T20) and for those genes from the intermediate induction cluster that show gradual induction. A group of genes from the intermediate induction cluster exhibited a maximal induction peak at T5 in the control, but seemed to have a delayed pattern of induction in the *TFIIS4* RNAi, reaching a maximal mRNA level only at T20. At this stage, however, closer examination of microarray expression patterns for individual genes is not possible since variations of the signals calculated from a single hybridization experiment are not statistically significant. Additional replicate experiments will be required to strengthen the statistical significance of our microarray data and to identify a potential set of genes with altered expression in *TFIIS4*-RNAi.

## Discussion

### TFIIS4 couples transcription and DNA elimination in *P*. *tetraurelia*

The functional analysis of TFIIS4 in *P*. *tetraurelia* established the role of a TFIIS homolog in the control of developmentally programmed DNA elimination. Our data indicate that TFIIS4 influences all kinds of genome rearrangements: it stimulates the precise excision of a large group of IESs, favors the elimination of multicopy transposons and the inheritance of macronuclear deletions. We show here that all three tested IESs are transcribed in a TFIIS4-dependent manner by the time DNA elimination takes place in the new MAC, and that IES transcription occurs before IES removal from the genome. It is therefore possible that TFIIS4 is necessary for transcription of all IESs–short and long, maternally or non-maternally controlled. Interestingly, only 48% of all IESs would then require TFIIS4-dependent transcription in order to be excised properly. TFIIS4-dependent IESs do not share any common features with regard to their sequence, end consensus or presence in coding or non-coding regions. Nevertheless, the dependence upon TFIIS4 seems to exhibit an IES size bias: excision of less than 30% of the shortest IESs (26–32 bp) requires the presence of TFIIS4 whereas up to ~60% of the IESs larger than 100 bp depend upon TFIIS4 for their excision. Our study, therefore, provides the first example of the participation of a TFIIS homolog in both the control of non-coding transcription and the regulation of programmed genome rearrangements.

Using genome-wide microarrays, we obtained no convincing evidence that TFIIS4 is involved in the induction of mRNA synthesis during sexual processes, even though we cannot completely exclude this possibility. Northern blot hybridization performed for two essential IES excision genes—*PGM* and *NOWA1/2* –confirmed that normal expression patterns are observed in *TFIIS4*-silenced cells. In particular, *PGM* mRNA displayed a wild-type “intermediate induction profile”, with a maximum induction peak around the time when IES excision starts (T5, see Fig 4A and 4B) and a decrease at later time-points (S9A Fig). This observation stands in contrast to previous work, which suggested that inhibition of genome rearrangements may cause dramatic mRNA accumulation for genes from the “intermediate induction cluster” [19,47]. In particular, *PGM* transcripts were found to accumulate in cells depleted for the essential Pgm partner Ku80c, suggesting the existence of a transcriptional feedback loop depending upon the completion of genome rearrangements. In contrast, no accumulation of *PGM* transcripts was observed at late autogamy time-points in the *TFIIS4* RNAi. This difference may indicate that none of the TFIIS4-dependent IESs is involved in the control of this putative transcriptional feedback loop.

### TFIIS4-dependent zygotic transcription and the model for RNA-mediated regulation of programmed genome rearrangements

**Fig 8:**
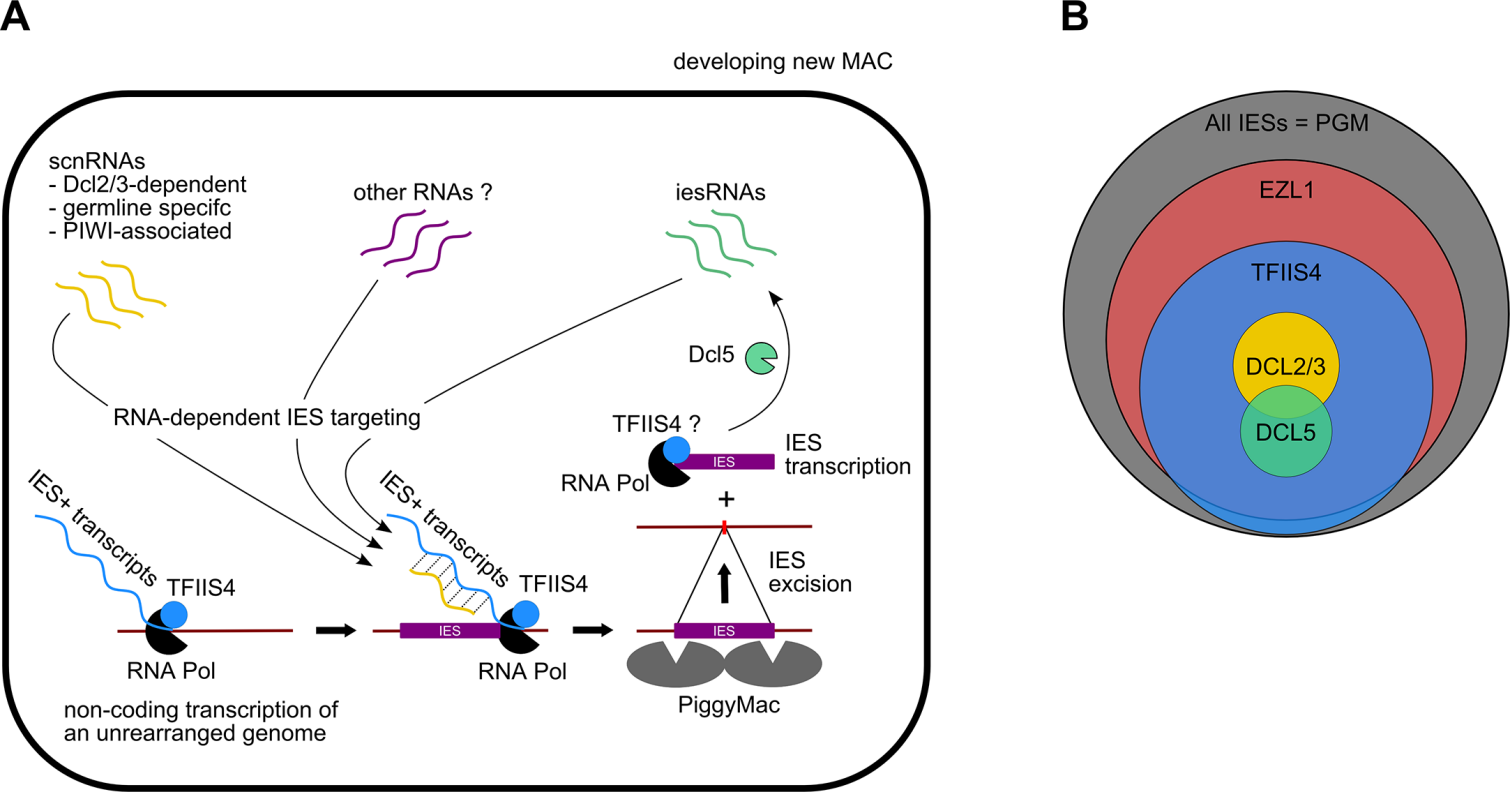
Proposed role of TFIIS4 in RNA-dependent DNA elimination. (A) Possible role of TFIIS4 in the new developing MAC. Description in the text. (B) Summary of the impact of *TFIIS4*, *EZL1*, *DCL2/3* or *DCL5* silencing of on IES excision. The area of each circle is proportional to the fraction of IESs that significantly depend on each factor.

The present study confirms the existence of IES-containing transcripts in *P*. *tetraurelia* and provides the first evidence that IES transcripts originate from the developing new MAC. Our work shows that *TFIIS4* mRNA starts to accumulate at early time-points of autogamy–as soon as meiotic cells can be detected. Yet, we note that the GFP fluorescence is still very weak at the stage and increases only in the new MACs. Two alternative explanations can be proposed for this delay: either TFIIS4 protein production is delayed relative to mRNA synthesis or protein is expressed but is diluted in the entire cell and cannot be detected. We cannot therefore definitely exclude the possibility that TFIIS4 plays a role in the processes that precede new MAC development, especially because some amounts of the GFP-TFIIS4 localize to the old MAC. For the moment, however, we found no evidence for its involvement in scnRNA synthesis or selection. We showed nevertheless that TFIIS4 is involved in synthesis of zygotic IES+ transcripts. We propose therefore that TFIIS4-dependent nascent zygotic transcripts are pairing substrates for IES-specific scnRNAs in the new MAC [13]. In the current version of the genome scanning model (Fig 8A), the 25-nt scnRNAs produced in meiotic MICs by the Dicer-like proteins Dcl2/3 [15,17] are transferred from the old MAC, in which they have become enriched for germline-specific sequences, to the developing new MAC, in which they are thought to pair to homologous nascent transcripts. According to the model, the pairing of scnRNAs to zygotic nascent transcripts leads to loading of chromatin modifications and, eventually, allows the targeting of DNA elimination. However, recent discoveries [17,19] indicate that the genome scanning model with a central role of scnRNAs does not explain the entire complexity of genome rearrangements in *Paramecium*. High-throughput analysis of IES retention after RNAi knock-down of particular genes allows us, nevertheless, to draw some conclusions regarding a possible interplay of TFIIS4 with other factors.

All IESs appear to give rise to scnRNAs to the same extent [17,48] and all are probably transcribed in a TFIIS4-dependent manner during MAC development (this work). The fact that nearly all IESs requiring scnRNAs (~3,200 Dcl2/3-dependent IESs) or iesRNAs (~3,000 Dcl5-dependent IESs) also depend upon TFIIS4 for their excision (Figs 4F and 8B), and the observation that these two partially overlapping IES subsets are among the most strongly dependent upon TFIIS4 (Table 2 and S11A and S11B Fig), is consistent with a model in which both types of sRNAs interact with TFIIS4-dependent IES+ nascent transcripts. However, the current genome scanning model, including scnRNAs and iesRNAs, explains the control of excision for only around 12% of all IESs, which is a few times less than those anticipated to be maternally controlled [43]. Thus, we cannot exclude that a novel class of sRNAs of yet unknown origin, which would be independent on Dicer-like proteins Dcl2/3 and Dcl5 (as proposed in [19]), interacts with TFIIS4-dependent zygotic transcripts to promote IES excision. Different types of sRNAs synthesized through Dicer-independent pathways were reported in other organisms, including the germline-specific piRNAs in metazoans (reviewed in [49]) or the disiRNAs of the filamentous fungus *Neurospora crassa*, all of which are associated with DNA methylation [50].

Practically all TFIIS4-dependent IESs are also dependent on Ezl1, a histone-methyl transferase required for excision of two-thirds of IES sequences [19]. Both proteins have the strongest impact on the longest IESs, which have a higher probability of being covered by a nucleosome. It may be a sign of some functional link between TFIIS4-dependent transcription and the mechanisms necessary for recognition of most (but not all) regions that need to be marked by H3K9/K27 trimethylation for their elimination. An alternative hypothesis would be that TFIIS4-dependent IES transcription plays another role, which would be unrelated to the pairing of sRNAs, for instance by opening up chromatin and allowing access of the Pgm complex to its target sites. This may be achieved by a similar mechanism to that reported for class switch recombination (CSR), through which the constant regions of antibodies are exchanged (reviewed in [51]): in this system, a nascent RNA synthesized at the recombination locus forms a hybrid RNA-DNA R-loop structure that displaces the complementary DNA strand, providing a recombination substrate. Non-coding transcription was also shown to have an impact on V(D)J recombination, which also plays a role in generating the diversity of the immune response. In this process, transcription is believed to affect recombination by altering chromatin structure (for review see [52]).

Finally, a large fraction of IESs (52%) do not require TFIIS4-dependent transcription to be excised properly. Most of these IESs are among the shortest ones, which appeared in the genome a relatively long time ago (see [9]). We suggest that these IESs have evolved to become independent of their own transcription for efficient excision–they give rise to scnRNA [17] and probably also to IES+ transcripts from the new MAC, but the presence of these RNAs is not necessary for their elimination. We may therefore consider a general model, in which foreign DNA sequences inserted recently in the germline genome, like transposable elements, are recognized and eliminated from the somatic genome using a specialized RNAi mechanism requiring sRNAs and TFIIS4-dependent zygotic nascent transcripts (as proposed in [13]). The physical elimination of foreign DNA depends on the PiggyMac domesticated transposase [12] and on components of the non-homologous end-joining (NHEJ) repair pathway [47,53] (discussed in [10,11]). Over time, these sequences would have evolved to yield IESs, by shortening in size and eventually becoming independent from the sRNA machinery. How current IESs are recognized and targeted for excision still remains an open issue, but deciphering the underlying molecular mechanisms will certainly provide a better understanding of other developmentally programmed chromatin diminution systems that were reported in numerous eukaryotes [54].

### A novel function in non-coding transcription for a specialized TFIIS factor

TFIIS is conserved in most eukaryotes and functional homologs are also found in *Archaea* (GreA and GreB proteins) and in some viral genomes [55]. In yeast, plants and worm, TFIIS factors are encoded by a single gene, while two genes can be found in the genomes of *Trypanosoma* and *Drosophila*. Three genes are present in vertebrate genomes [56], which may be explained by whole-genome duplications that played an important role in vertebrate evolution [57]: retention of these three copies was proposed to be correlated with high organism complexity. During evolution, four TFIIS families have emerged in *Paramecium*, independently from the multigene *TFIIS* families found in other eukaryotic species [58], including the ciliate *Oxytricha trifallax* (S2 Fig). All *P*. *tetraurelia* proteins contain the three canonical TFIIS domains and represent the most divergent group of TFIIS factors encoded by a single genome. This study shows, for the first time in a unicellular organism, that TFIIS factors may be specialized with regard to their expression patterns and localization, even though future in-depth studies will be required to unravel their exact respective functions. TFIIS1 and TFIIS3 seem to be linked to expression of the somatic genome, while TFIIS2 and TFIIS3 might be required for general transcription of the germline genome during meiosis. Finally, TFIIS4 is specifically expressed during sexual processes and is responsible for zygotic non-coding transcription, therefore playing an essential role during MAC development and assembly of the new somatic genome.

Interestingly, according to currently available studies, the developmentally programmed activation of *TFIIS* gene expression seems to be a general rule in ciliates. Up-regulation of gene expression during conjugation was reported for *conN1* in *Moneuplotes crassus* [59] and the TFIIS-encoding *TTHERM_00691200* in *Tetrahymena thermophila* [60]. The strong phenotype observed in a *TFIIS4* RNAi is reminiscent of the embryonic lethality of a *TCEA1* KO in mouse [61] and of the function of TCEA3, which is highly enriched in mouse embryonic stem cells and regulates their pluripotent differentiation [62]. Thus, *Paramecium* provides a promising system for the functional analysis of TFIIS function during development. Execution of developmental programs in eukaryotes involves several ncRNAs and involves epigenetic programming of the genome (reviewed in [63]). Our work on TFIIS4 in *Paramecium* demonstrates, for the first time, a role of a TFIIS homolog as an essential factor for the production of regulatory non-coding transcripts, and establishes a novel connection between non-coding transcription and the control of genome plasticity.

## Materials and Methods

### *Paramecium* strains, cultivation and autogamy

All experiments were carried out with *Paramecium tetraurelia* strain 51new [64]. In large-scale silencing experiments, a 51 ∆*A* ∆*ND7* strain carrying an injection-induced macronuclear deletion of the surface antigen *A* gene [42] and a silencing-induced macronuclear deletion of the *ND7* gene [45] was used. In microinjection experiments, strain 51 *nd7*-*1* was used as described previously [47].

*Paramecium* cell cultivation and autogamy were carried out as described previously at 27°C [65]. For standard cultivation, cells were grown in a Wheat Grass Powder medium (WGP, Pines International, Lawrence, KS, USA) inoculated the day before with *Klebsiella pneumoniae*, and supplemented just before use with 0.8 μg/ml β-sitosterol (Merck) [66].

### DNA and RNA extraction

Genomic DNA and total RNA were extracted from ~400,000 *Paramecium* cells during vegetative growth and at different time points of the autogamy time-course, as described in [12].

### Northern and Southern blot hybridization

For northern blots, 20 μg of denatured total RNA were loaded on a 1% agarose gel. Electrophoresis, blotting and hybridization were performed as described previously [40], or using the NorthernMax-Gly Kit (Ambion) as recommended by the supplier. Southern blot hybridization was performed as in [12]. Electrophoresis of *PstI*-digested genomic DNA (2 μg per lane) or RT-PCR products were carried out in 0.8%–2% agarose gels (Resolva GQT–for smaller products, Basica LE GQT for larger fragments (Prona)) in 0.5x TBE buffer, and transferred to Hybond N+ or Hybond XL membranes (GE Healthcare) in 0.4 N NaOH. Double-stranded probes were labeled by random priming with [α-^32^P] dATP (3000 Ci/mmol, Hartmann Analytic). Oligonucleotide probes were labeled with [γ-^32^P] ATP (3000 Ci/mmol, Hartmann Analytic) using T4 polynucleotide kinase. Southern blots were hybridized at 60°C and washed in 0.2x SSC and 0.1% SDS at 60°C prior to image plate exposure. Northern blots were hybridized at 42°C in Ultrahyb buffer (Ambion) and washed as recommended by the supplier. All radioactive signals were quantified using ImageJ. Hybridization probes are described in S1 and S2 Tables.

### Construction of GFP fusions

Plasmid pGFP-TFIIS4-e encoding an N-terminal GFP fusion to TFIIS4 was constructed by inserting a 1294-bp fragment containing the *TFIIS4* open reading frame and its potential 179-bp terminator region (bp 151716.150423 from accession number NW_001799642.1) between the *BamH*I and *Pst*I sites of plasmid pZCΔRIX (kindly provided by E. Meyer & S. Malinsky), directly downstream of the EGFP coding sequence optimized for *Paramecium* codon usage [44]. Then, the putative promoter of *TFIIS4* (bp 151832.151714) was inserted between the *Sal*I and *Xba*I sites of the plasmid. The other N-terminal GFP fusions (pGFP-TFIIS1a, pGFP-TFIIS1c, pGFP-TFIIS2a, pGFP-TFIIS2b, pGFP-TFIIS3) were obtained by an overlapping PCR method [67]. In general, each construct contained the putative promoter, coding sequence and putative terminator region of the appropriate *TFIIS* gene (exact coordinates of cloned genomic fragments are given in S1 Table). For each construct, DNA fragments representing the endogenous promoter, the EGFP coding sequence and the *TFIIS* coding sequence with its putative terminator region were amplified separately. Each PCR product was designed to contain a 50-bp overlap with its adjacent fragment(s), so that all fragments could hybridize in the proper order to assemble the desired sequence. Annealed fragments were amplified with external primers containing overhangs with restriction sites, and then cloned between the *Xho*I and *Pst*I sites of the pCRscript vector (Invitrogen). Platinum Taq polymerase (Invitrogen) was used in all PCR reactions and at each step PCR products were purified using the Invisorb Fragment CleanUp kit (Stratec). All constructs were checked by Sanger sequencing (IBB, PAS).

### Injection of GFP fusion transgenes

Before microinjection, all plasmids were purified using a QIAfilter Plasmid Maxi Kit (Qiagen) and linearized within the vector sequence. They were filtered through a 0.22 μm Ultrafree-MC filter (Millipore), precipitated with ethanol and dissolved in filtered water to a final concentration of 5 μg/μL. Linearized plasmids carrying GFP fusion transgenes were microinjected into the MAC of vegetative 51 *nd7*-*1* cells, as described previously [44]. Briefly, *Paramecium* cells were microinjected in Dryl solution containing 0.2% bovine serum albumin, under a paraffin oil film, while they were visualized with a phase-contrast inverted microscope. All observations were performed using a Nikon Eclipse E800 or a Zeiss Axioplan 2 epifluorescence microscope.

### Gene inactivation by RNAi

All RNAi plasmids are derivatives of vector L4440 [68] and carry a fragment of the target gene inserted between two convergent T7 promoters (inserts used in this study are listed in S1 Table). Additionally, the *PiggyMac* RNAi plasmid PGM-1 [12] was used. Control RNAi plasmids were: p0ND7c [45] and pICL7a [69], which target the non-essential *ND7* and *ICL7a* genes, respectively. In all feeding experiments, the efficiency of *ND7* silencing was confirmed by the lack of trichocyst discharge in the presence of picric acid. *ICL7* silencing was checked by transferring cells in AED 0.5% buffer containing Ca^2+^, and observation of a failure in cell shortening and backward swimming behavior [70]. Silencing media were prepared basically as described in [71] and [40], by inoculating precultures of the appropriate bacterial strains into WGP medium containing 0.1 mg/mL ampicillin. Following 6–8 hrs of shaking at 37°C, bacterial cultures were diluted six-fold into the same medium containing 0.4 mM IPTG to induce dsRNA synthesis. After overnight induction at 37°C, all silencing media were supplemented with 0.8 μg/mL β-sitosterol (Merck) before use.

### Microarray analysis of gene expression

Five samples (V, St, T0, T5, T10 and T20) from a *TFIIS4* silencing and a control (*ICL7* silencing) autogamy time-course experiment were selected and sent to PartnerChip (Evry, France) for cDNA labeling and hybridization on NimbleGen (Roche Nimblegen, Madison, WI) microarrays “101018_Paramecium_L_EXP” (GEO no. GPL18944, SET01). The data were processed and normalized as previously described [28] (GEO no. GSE64682).

### Cell lysis and purification of new developing macronuclei

As described in [9], a fraction enriched in late new developing macronuclei was obtained through different centrifugation steps from 3.8 L of autogamous cells (at a concentration of ~2000 cells/ml) submitted to *TFIIS4* RNAi in the independent experiment from the one used for PCR assays. After dialysis, 4.4 μg of DNA was obtained. Southern-blot detection of the retention of IES 51G4404 was performed by ^32^P-labelling of the Gmac probe [12], which corresponds to MAC sequences just downstream of IES 51G4404 within the surface antigen *G* ^*51*^ gene. The contamination with bacterial DNA was estimated by hybridization of the same blot with a ^32^P-labelled 23S rDNA probe from *K*. *pneumoniae* [9].

### Genome-wide analysis of IES retention

The DNA obtained from the nuclear fraction enriched for late developing MACs was submitted to paired-end sequencing using an Illumina HiScan SQ next-generation sequencer. The average shotgun library fragment length was 250 bp and the read length equaled 101 nt (GenBank Sequence Read Archive SRP047508). After quality filtering and removal of adapters, Illumina reads were processed as described elsewhere [48], and aligned to the reference genomes (*P*. *tetraurelia* MAC reference genome and MAC+IES reference genome) using BWA [72] with default parameters. Alignments were indexed with Samtools [73].

For each sample, IES retention scores (RS) were determined as described in [19]. For each IES that was previously identified in [9], the number of reads that contain the IES sequence (symbolized IES+) and the number of reads that contain only the macronuclear IES excision junction consisting of a TA dinucleotide (IES-) were determined. Only reads with unambiguous alignments were counted. Each read was counted only once to avoid over-counting owing to paralogous matches. Reads were only counted at IES ends, to avoid length biases resulting from IES length variation. The fraction of IES+ reads/(IES+ and IES-) reads gives the RS.

Then, we compared the RS of a given IES to the control RS observed for the control DNA sequencing (as described in [19]) to make sure that the observed retention can be attributed to *TFIIS4* silencing. First we calculated the confidence interval (alpha = 0.95) of the control RS value, using the Pearson-Klopper exact method as implemented by the R binom package version 1.0–5 [74]. Then we tested for higher retention in the experiment, thanks to a frequency comparison test (based on a binomial law of probability) between the experimental RS and the upper bound of the confidence interval in the control. Resulting p-values were adjusted for multiple testing using the Benjamini &Hochberg method [75]. IESs for which the frequency comparison test gives an adjusted p-value lower than 0.05 are considered significantly retained in the sample.

### sRNA sequencing

Total RNA samples were run on a denaturing 15% polyacrylamide-urea gel. After electrophoresis the gel was stained with SYBR Gold (Invitrogen) and 20–30 nt RNA fraction was cut from the gel. Sequencing libraries were prepared using oligonucleotides from TruSeq Small RNA Sample Prep Kit (Illumina). NextSeq 500 (Illumina) reads (SRX1022957) were trimmed to extract small RNA sequences between 20 and 30 nt. Subsequently, reads matching to rDNA, genomes of food bacteria (*K*. *pneumoniae*, *E*. *coli*), mitochondrial genome and L4440 vector sequence were removed using BWA (v0.7.8-r455) [72] (allowing 1 mismatch). Filtered reads were mapped consecutively on the MAC genome and the IESs from the MAC+IES reference using BWA (allowing no mismatches and matching on a unique location). We used the total number of reads mapped on a *Paramecium* reference (MAC or IES) to normalize the counts.

### Reference genomes

The following reference genomes [9] were used in the IES analyses and for read mapping:

MAC reference (strain 51):

http://paramecium.cgm.cnrs-gif.fr/download/fasta/assemblies/ptetraurelia_mac_51.fa

MAC+IES reference (strain 51):

http://paramecium.cgm.cnrs-gif.fr/download/fasta/assemblies/ptetraurelia_mac_51_with_ies.fa

PGM contigs:

http://paramecium.cgm.cnrs-gif.fr/download/fasta/assemblies/ptetraurelia_PGM_k51_ctg.fa

Macronuclear DNA reads for PiggyMac-depleted cells [9], Ezl1-depleted cells and control DNA-seq [19], Dcl2/3-depleted cells and Dcl5-depleted cells [17] were obtained from the European Nucleotide Archive (Accession number ERA137420, ERA309409) and the GenBank Sequence Read Archive (Accession number SRX387766, SRX387766), respectively.

### RT–PCR detection of non-coding RNAs

Total RNA samples were treated with RNase-free DNaseI (Ambion) for 30 min at 37°C, then extracted with acid phenol pH 4.3 (Sigma) and precipitated with ethanol. Five μg of RNA was reversed-transcribed using RevertAid H Minus Reverse Transcriptase (Thermo Scientific) according to the supplier’s instructions, using random hexameric primers (Thermo Scientific). IES-specific PCR primers were designed to amplify fragments of the maternally controlled IESs 51G4404 and 51A6649, as well as the non-maternally controlled IES 51A4404. Conditions of PCR amplification using DreamTaq DNA Polymerase (Thermo Scientific) were adjusted in order not to saturate the amplification reactions, which were subsequently blotted and visualized by Southern blot hybridization using specific IES probes. Normalization was performed relative to the cDNA of the constitutively expressed *T1b* gene, which encodes a trichocyst matrix protein (TMP1b). Oligonucleotide sequences are listed in S2 Table.

## Supporting Information

S1 Fig Alignment and conservation of predicted structural domains for *P*. *tetraurelia* TFIIS proteins. Full protein sequences were aligned using T-Coffee [77] with default parameters and corrected manually. The alignment was colored using Boxshade at http://www.ch.embnet.org/software/BOX_form.html (grey: similar residues; black: identical residues; fraction of aligned residues that must agree for shading: 0.4). Only regions encompassing the conserved TFIIS domains are shown, since the region between domains I and II did not give significant alignment. The structural annotation below the alignment (structural features of the protein represented by filled rectangles) is based on the structure of *S*. *cerevisiae* Dst1p [21,32]. The Zn finger-forming conserved cystein residues and the DE dipeptide are highlighted in green and pink, respectively. The secondary structure prediction for *P*. *tetraurelia* TFIIS proteins was run using PSIPRED from the PRALINE package [78]: red and yellow open rectangles indicate the prediction of alpha helices in domains I and II or in the linker region, respectively. Blue open rectangles designate predicted beta-strands in domain III. Similar prediction results were obtained using NPS@ [79]. Abbreviations and accession numbers are as follows: Pt: *Paramecium tetraurelia*—accession numbers as in the legend of Fig 1A; Tt: *Tetrahymena thermophila* TFIIS—XP_001032085.3; Ot: *Oxytricha trifallax* 22233_0_g55—Contig22233_0_g55(protein), 1015_0_g5—Contig1015_0_g5(protein), 14486_0_g34—Contig14486_0_g34(protein) (OxyDB); Mc: *Moneuplotes crassus* conN1—AAG00939; Im: *Ichthyophthirius multifiliis*—IMG5_116810 (IchDB); Lm: *Leishmania major* Lm-TFIIS1-1—CAJ04034, Lm-TFIIS2-1—CAJ06790; Tb: *Trypanosoma brucei* TFIIS1—XP_828571, TFIIS2-1—XP_951597; Hs: *Homo sapiens* TCEA1—NP_006747, TCEA2—NP_003186, TCEA3—NP_003187; At: *Arabidopsis thaliana* TFIIS—NP_181390; Ce: *Caenorhabditis elegans* TFIIS—NP_495941; Dm: *Drosophila melanogaster* TFIIS—NP_476967.1, CG8117—NP_573049.2; Sp: *Schizosaccharomyces pombe* tfs1—CAC19733; Sc: *Saccharomyces cerevisiae* Dst1—NP_011472.1.
(PDF)

S2 Fig Neighbor-joining tree of TFIIS proteins from *Paramecium* genus and other ciliates. The evolutionary history was reconstructed as described in Fig 1 legend.
(TIFF)

S3 Fig Northern blot validation of expression profiles for all TFIIS-encoding genes. (A) Histograms show the progression of autogamy in strain 51new mt8 grown on standard *K*. *pneumoniae* medium. For each time-point (V: vegetative culture; -4: meiosis; 0: around 50% of cells with fragmented MAC; 5 to 64: 5 to 64 hours following time 0, respectively), cells were stained with DAPI to visualize old and new MACs. V: vegetative parental MAC; M: meiosis; S: skein formation; F: fragmented old MAC but no detectable developing new MACs; A: fragmented old MAC + 2 visible anlagen, C: post-karyonidal cells. (B) Northern blots and histograms showing the validation of expression profiles for each *TFIIS* gene. Two identical blots were used in parallel for the successive hybridization of individual ^32^P-labelled gene probes. Details for all hybridization probes are listed in S1 Table. Blot 1 was used for *TFIIS1a*, *TFIIS1c*, *TFIIS3* and *TFIIS4*. Blot 2 was used for *TFIIS2a* and *TFIIS2b*. Hybridization signals were normalized using 17S rRNA. Hybridization of ^32^P-labelled 17S rDNA probe with each blot is shown at the bottom of the figure.
(TIFF)

S4 Fig Localization of GFP fusion proteins for TFIIS1c and TFIIS2b. Panels a and f show vegetative cells. All other panels show successive stages of autogamy: panels b, g, h and i–meiosis: panel g–meiotic crescent stage; panel b–cell after meiosis I; panel h–cells with 8 haploid nuclei resulting from meiosis II; panels c and i–cells with fragmented old MAC; panels d and j–early MAC development; panels e and k–late MAC development. All arrows/arrowheads as in Fig 2. In panels c and d, the asterisks denotes additional fluorescent signal observed with the GFP filter due to the presence of crystals in the cytoplasm. (A) A GFP-TFIIS1c fusion localizes to old, then new MACs. (B) A GFP-TFIIS2b fusion localizes to old, then new MACs and is present in meiotic MICs. The GFP-TFIIS2b fusion shows a stronger signal in vegetative cells (panel f) than GFP-TFIIS2a—it might be explained by a higher copy number of the injected transgene, which may cause overexpression of the protein.
(TIFF)

S5 Fig Cytological observation of DAPI-stained cells silenced for *TFIIS4* and control gene (*ICL7*). (TIFF)

S6 Fig Autogamy time-course of cells silenced for *TFIIS4* relative to a control RNAi. (A) Histograms show the progression of autogamy in strain 51mt8 *∆A ∆ND7*. As a control we used cells silenced for the *ICL7* unrelated gene. For each time-point (V: vegetative culture; -2.5: meiosis and early MAC fragmentation; 0: around 50% of cells with fragmented MAC; 5 to 56: 5 to 56 hours following time 0, respectively), cells were stained with DAPI to visualize old and new MACs. V: vegetative parental MAC; M: meiosis; S: skein formation; F: fragmented old MAC but no detectable developing new MACs; A: fragmented old MAC + 2 visible anlagen, C: post-karyonidal cells. (B) Northern blot validation of *TFIIS4* silencing. The blot was hybridized sequentially with a *TFIIS4* probe and 17S rRNA probe as a control of RNA loading. Histograms present expression of *TFIIS4* during autogamy after normalization. In the latest time-points, transcripts corresponding to the gene are no longer efficiently down-regulated–probably due to lower amount of siRNA present in the cells after long starvation period. This may explain some residual survival in post-autogamous cells and delayed partial excision of some IESs.
(TIFF)

S7 Fig Relation between TFIIS4 retention scores, EZL1 retention scores and IES size. (A) The heatmap shows the relation between EZL1 retention score and TFIIS4 retention score for IESs that are significantly retained in *TFIIS4* RNAi. The color represents the number of IESs according to the legend on the right. (B) Each group of IESs corresponds to a peak in the periodic IES size distribution [9]. The box plot displays the TFIIS4 IES retention score distribution for each group. The median retention score (horizontal line inside the box) and the first (top of box) and third (bottom of box) quartiles are shown. Stars beneath the median indicate that the retention score distribution of a given group is significantly different from the retention score distribution of the previous group according to a Mann-Whitney test. The median retention score significantly increases between the groups of small IESs (<82 bp), indicating that excision of the smallest IESs is mostly independent of TFIIS4 expression. For larger IESs the median increases slowly but the retention score distribution is significantly different only for IESs larger than 200 bp and, similarly to EZL1 and DCL2/3 [17,19], for the largest IESs (> 1 kb).
(TIFF)

S8 Fig Detection of non-coding RNAs in *TFIIS4*-silenced cells during autogamy. (A) Southern blot detection of *G*-gene transcripts obtained in RT-PCR reaction using primers located within the macronuclear sequences flanking IES 51G4404 (see S6 Fig, panel A for details about autogamy stages). PCR reactions were performed with the same set of first strand cDNA as used in IES+ transcript detection. PCR products (279 bp) correspond to IES-free (IES-) maternal transcripts. For each sample, the lane marked as “-” presents the control without reverse transcriptase. Lanes-*RNA* and-*PCR* are negative controls without RNA, *ctrl* corresponds to positive control performed on genomic DNA. (B) Histograms showing the progression of autogamy in a control culture (*ND7*-silenced cells) and in *PiggyMac*-silenced cells (strain 51new mt8) [47]. For details see legend to S6 Fig. (C) Histograms show normalized number of sRNAs that match to the *Paramecium* genome for the biological replicate of the experiment shown in Fig 7B. Details about autogamy stages are shown in S8 Fig, panel D. (D) Histograms show the progression of autogamy in strain 51mt8 *∆A ∆ND7* –biological replicate of the experiment used for most of the analysis. Cells were silenced for the *TFIIS4* and *ICL7* unrelated gene. For details see legend to S6 Fig.
(TIFF)

S9 Fig Coding transcription. (A) Northern blot hybridization of RNA obtained in an autogamy time-course experiment (see S6 Fig), using *PiggyMac* and *NOWA1/2* probes. 17S rRNA probe was used as a loading control. (B) Microarray hybridization data obtained using the same RNA samples as in panel A. Gene expression heatmap was plotted using the previously obtained hierarchical clusterization of the set of 2467 genes that are most differentially expressed during autogamy [28]. The plot displays the samples both for *TFIIS4*-silencing and the control (*ICL7*-silencing) as columns, and the genes as rows. The color code goes from dark blue for the lowest normalized expression level to dark red for the highest expression level.
(TIFF)

S10 Fig Localization of a PiggyMac-GFP fusion in TFIIS4-depleted cells. Previously described PiggyMac-GFP fusion construct was used in this study [10]. The efficiency of *TFIIS4* silencing was confirmed by the 92% lethality observed in the sexual progeny. Control cells were silenced for the *ICL7* gene. Vertical panels show cells at different stages of MAC development–after 2, 3 and 4 days of starvation, respectively. White arrowheads indicate new MACs.
(TIFF)

S11 Fig Comparison of IES retention between *TFIIS4*, *DCL2/3* and *DCL5* RNAi. (A) Superimposed histogram of *TFIIS4* retention scores for all IESs (dark blue) and IESs retained following *DCL2/3* RNAi (yellow). Retention scores for IESs that are significantly retained in a *TFIIS4* RNAi are in light blue. (B) As in (A) for Dcl5-dependent IESs (green).
(TIFF)

S1 Table Coordinates of sequences used in this study as northern probes, inserts in GFP constructs and silencing constructs. (PDF)

S2 Table Oligonucleotides used in the study. (PDF)

S3 Table Sequence complexity of control, PGM and TFIIS4 datasets. This table shows the sequence complexity of PGM, TFIIS4 and the control (wild-type genome) datasets, using the contigs assembled from the PGM dataset as a reference. Sequencing reads were mapped on the entire reference, and coverage of each contig was determined in RPKM (reads per kilobase (kb) of contig per million mapped reads in the library). We consider that a contig is covered if its coverage is above 2 RPKM. The “PGM” reference contains contigs larger than 1 kb and covered by the PGM dataset. The “PGM not Control” contains contigs larger than 1 kb, covered by the PGM dataset but not by the control dataset, representing the MIC restricted regions, not collinear with the MAC. Each column indicates sum of the lengths of contigs covered by the given dataset.
(PDF)

## References

[1] Hirose T, Mishima Y, Tomari Y. Elements and machinery of non-coding RNAs: toward their taxonomy. EMBO Rep. 2014;15: 489–507. 10.1002/embr.201338390 24731943 PMC4210095

[2] Kung JT, Colognori D, Lee JT. Long noncoding RNAs: past, present, and future. Genetics. 2013;193: 651–669. 10.1534/genetics.112.146704 23463798 PMC3583990

[3] Tragante V, Moore JH, Asselbergs FW. The ENCODE Project and Perspectives on Pathways. Genet Epidemiol. 2014;38: 275–280. 10.1002/gepi.21802 24723339

[4] Borchert GM, Lanier W, Davidson BL. RNA polymerase III transcribes human microRNAs. Nat Struct Mol Biol. 2006;13: 1097–1101. 17099701 10.1038/nsmb1167

[5] Struhl K. Transcriptional noise and the fidelity of initiation by RNA polymerase II. Nat Struct Mol Biol. 2007;14: 103–105. 17277804 10.1038/nsmb0207-103

[6] Ernst C, Morton CC. Identification and function of long non-coding RNA. Front Cell Neurosci. 2013;7: 168 10.3389/fncel.2013.00168 24106460 PMC3788346

[7] Wierzbicki AT. The role of long non-coding RNA in transcriptional gene silencing. Curr Opin Plant Biol. 2012;15: 517–522. 10.1016/j.pbi.2012.08.008 22960034

[8] Duharcourt S, Lepère G, Meyer E. Developmental genome rearrangements in ciliates: a natural genomic subtraction mediated by non-coding transcripts. Trends Genet. 2009;25: 344–350. 10.1016/j.tig.2009.05.007 19596481

[9] Arnaiz O, Mathy N, Baudry C, Malinsky S, Aury JM, Wilkes CD, et al The Paramecium germline genome provides a niche for intragenic parasitic DNA: evolutionary dynamics of internal eliminated sequences. PLoS Genet. 2012;8 10.1371/journal.pgen.1002984 PMC3464196 23071448

[10] Dubois E, Bischerour J, Marmignon A, Mathy N, Regnier V, Betermier M. Transposon Invasion of the Paramecium Germline Genome Countered by a Domesticated PiggyBac Transposase and the NHEJ Pathway. Int J Evol Biol. 2012;2012: 436196 10.1155/2012/436196 22888464 PMC3408717

[11] Betermier M, Bertrand P, Lopez BS. Is non-homologous end-joining really an inherently error-prone process? PLoS Genet. 2014;10: e1004086 10.1371/journal.pgen.1004086 24453986 PMC3894167

[12] Baudry C, Malinsky S, Restituito M, Kapusta A, Rosa S, Meyer E, et al PiggyMac, a domesticated piggyBac transposase involved in programmed genome rearrangements in the ciliate Paramecium tetraurelia. Genes Dev. 2009;23: 2478–2483. 10.1101/gad.547309 19884254 PMC2779751

[13] Coyne RS, Lhuillier-Akakpo M, Duharcourt S. RNA-guided DNA rearrangements in ciliates: Is the best genome defense a good offense? Biol Cell. 2012;104: 309–325. 10.1111/boc.201100057 22352444

[14] Fang W, Wang X, Bracht JR, Nowacki M, Landweber LF. Piwi-interacting RNAs protect DNA against loss during Oxytricha genome rearrangement. Cell. 2012;151: 1243–1255. 10.1016/j.cell.2012.10.045 23217708 PMC3678556

[15] Lepère G, Nowacki M, Serrano V, Gout JF, Guglielmi G, Duharcourt S, et al Silencing-associated and meiosis-specific small RNA pathways in Paramecium tetraurelia. Nucleic Acids Res. 2009;37: 903–915. 10.1093/nar/gkn1018 19103667 PMC2647294

[16] Singh DP, Saudemont B, Guglielmi G, Arnaiz O, Gout JF, Prajer M, et al Genome-defence small RNAs exapted for epigenetic mating-type inheritance. Nature. 2014;509: 447–452. 10.1038/nature13318 24805235

[17] Sandoval PY, Swart EC, Arambasic M, Nowacki M. Functional Diversification of Dicer-like Proteins and Small RNAs Required for Genome Sculpting. Dev Cell. 2014;28: 174–188. 10.1016/j.devcel.2013.12.010 24439910

[18] Lepère G, Bétermier M, Meyer E, Duharcourt S. Maternal noncoding transcripts antagonize the targeting of DNA elimination by scanRNAs in Paramecium tetraurelia. Genes Dev. 2008;22: 1501–1512. 10.1101/gad.473008 18519642 PMC2418586

[19] Lhuillier-Akakpo M, Frapporti A, Denby Wilkes C, Matelot M, Vervoort M, Sperling L, et al Local Effect of Enhancer of Zeste-like Reveals Cooperation of Epigenetic and Cis-acting Determinants for Zygotic Genome Rearrangements. PLoS Genet. 2014;10: e1004665 10.1371/journal.pgen.1004665 25254958 PMC4177680

[20] Bouhouche K, Gout JF, Kapusta A, Bétermier M, Meyer E. Functional specialization of Piwi proteins in Paramecium tetraurelia from post-transcriptional gene silencing to genome remodelling. Nucleic Acids Res. 2011;39: 4249–4264. 10.1093/nar/gkq1283 21216825 PMC3105430

[21] Kettenberger H, Armache KJ, Cramer P. Architecture of the RNA polymerase II-TFIIS complex and implications for mRNA cleavage. Cell. 2003;114: 347–357. 12914699 10.1016/s0092-8674(03)00598-1

[22] Adelman K, Lis JT. Promoter-proximal pausing of RNA polymerase II: emerging roles in metazoans. Nat Rev Genet. 2012;13: 720–731. 10.1038/nrg3293 22986266 PMC3552498

[23] Ishibashi T, Dangkulwanich M, Coello Y, Lionberger TA, Lubkowska L, Ponticelli AS, et al Transcription factors IIS and IIF enhance transcription efficiency by differentially modifying RNA polymerase pausing dynamics. Proc Natl Acad Sci USA. 2014;111: 3419–3424. 10.1073/pnas.1401611111 24550488 PMC3948276

[24] Guglielmi B, Soutourina J, Esnault C, Werner M. TFIIS elongation factor and Mediator act in conjunction during transcription initiation in vivo. Proc Natl Acad Sci U S A. 2007;104: 16062–16067. 10.1073/pnas.0704534104 17901206 PMC2042162

[25] Kim B, Nesvizhskii AI, Rani PG, Hahn S, Aebersold R, Ranish JA. The transcription elongation factor TFIIS is a component of RNA polymerase II preinitiation complexes. Proc Natl Acad Sci U S A. 2007;104: 16068–16073. 10.1073/pnas.0704573104 17913884 PMC2042163

[26] Ghavi-Helm Y, Michaut M, Acker J, Aude JC, Thuriaux P, Werner M, et al Genome-wide location analysis reveals a role of TFIIS in RNA polymerase III transcription. Genes Dev. 2008;22: 1934–1947. 10.1101/gad.471908 18628399 PMC2492739

[27] Carrière L, Graziani S, Alibert O, Ghavi-Helm Y, Boussouar F, Humbertclaude H, et al Genomic binding of Pol III transcription machinery and relationship with TFIIS transcription factor distribution in mouse embryonic stem cells. Nucleic Acids Res. 2012;40: 270–283. 10.1093/nar/gkr737 21911356 PMC3245943

[28] Arnaiz O, Goût JF, Bétermier M, Bouhouche K, Cohen J, Duret L, et al Gene expression in a paleopolyploid: a transcriptome resource for the ciliate Paramecium tetraurelia. BMC Genomics. 2010;11: 547 10.1186/1471-2164-11-547 20932287 PMC3091696

[29] Arnaiz O, Sperling L. ParameciumDB in 2011: new tools and new data for functional and comparative genomics of the model ciliate Paramecium tetraurelia. Nucleic Acids Res. 2011;39: 632–636. 10.1093/nar/gkq918 PMC3013783 20952411

[30] Nakanishi T, Nakano A, Nomura K, Sekimizu K, Natori S. Purification, gene cloning, and gene disruption of the transcription elongation factor S-II in Saccharomyces cerevisiae. J Biol Chem. 1992;267: 13200–13204. 1618824

[31] Aury JM, Jaillon O, Duret L, Noel B, Jubin C, Porcel BM, et al Global trends of whole-genome duplications revealed by the ciliate Paramecium tetraurelia. Nature. 2006;444: 171–178. 10.1038/nature05230 17086204

[32] Booth V, Koth CM, Edwards AM, Arrowsmith CH. Structure of a conserved domain common to the transcription factors TFIIS, elongin A, and CRSP70. J Biol Chem. 2000;275: 31266–31268. 10.1074/jbc.M002595200 10811649

[33] Olmsted VK, Awrey DE, Koth C, Shan X, Morin PE, Kazanis S, et al Yeast transcript elongation factor (TFIIS), structure and function. I: NMR structural analysis of the minimal transcriptionally active region. J Biol Chem. 1998;273: 22589–22594. 9712887 10.1074/jbc.273.35.22589

[34] Jeon C, Yoon H, Agarwal K. The transcription factor TFIIS zinc ribbon dipeptide Asp-Glu is critical for stimulation of elongation and RNA cleavage by RNA polymerase II. Proc Natl Acad Sci U S A. 1994;91: 9106–9110. 8090778 10.1073/pnas.91.19.9106 PMC44756

[35] Awrey DE, Shimasaki N, Koth C, Weilbaecher R, Olmsted V, Kazanis S, et al Yeast transcript elongation factor (TFIIS), structure and function. II: RNA polymerase binding, transcript cleavage, and read-through. J Biol Chem. 1998;273: 22595–22605. 9712888 10.1074/jbc.273.35.22595

[36] McGrath CL, Gout JF, Doak TG, Yanagi A, Lynch M. Insights into Three Whole-Genome Duplications Gleaned from the Paramecium caudatum Genome Sequence. Genetics. 2014;197: 1412–1428. 10.1534/genetics.114.163287 PMC4125410 24840360

[37] McGrath CL, Gout JF, Johri P, Doak TG, Lynch M. Differential retention and divergent resolution of duplicate genes following whole-genome duplication. Genome Res. 2014; 10.1101/gr.173740.114 PMC4199370 25085612

[38] Berger JD. Nuclear differentiation and nucleic acid synthesis in well-fed exconjugants of Paramecium aurelia. Chromosoma. 1973;42: 247–268. 4354261 10.1007/BF00284774

[39] Galvani A, Sperling L. RNA interference by feeding in Paramecium. Trends Genet. 2002;18: 11–12. 11750689 10.1016/s0168-9525(01)02548-3

[40] Nowak JK, Gromadka R, Juszczuk M, Jerka-Dziadosz M, Maliszewska K, Mucchielli MH, et al Functional study of genes essential for autogamy and nuclear reorganization in Paramecium. Eukaryot Cell. 2011;10: 363–372. 10.1128/EC.00258-10 21257794 PMC3067474

[41] Williams LA, Kane CM. Isolation and characterization of the Schizosaccharomyces pombe gene encoding transcript elongation factor TFIIS. Yeast. 1996;12: 227–236. 10.1002/(SICI)1097-0061(19960315)12:3<227::AID-YEA905>3.0.CO;2–9 8904334

[42] Gratias A, Lepère G, Garnier O, Rosa S, Duharcourt S, Malinsky S, et al Developmentally programmed DNA splicing in Paramecium reveals short-distance crosstalk between DNA cleavage sites. Nucleic Acids Res. 2008;36: 3244–3251. 10.1093/nar/gkn154 18420657 PMC2425466

[43] Duharcourt S, Keller AM, Meyer E. Homology-dependent maternal inhibition of developmental excision of internal eliminated sequences in Paramecium tetraurelia. Mol Cell Biol. 1998;18: 7075–7085. 9819394 10.1128/mcb.18.12.7075 PMC109289

[44] Nowacki M, Zagorski-Ostoja W, Meyer E. Nowa1p and Nowa2p: novel putative RNA binding proteins involved in trans-nuclear crosstalk in Paramecium tetraurelia. Curr Biol. 2005;15: 1616–1628. 10.1016/j.cub.2005.07.033 16169483

[45] Garnier O, Serrano V, Duharcourt S, Meyer E. RNA-mediated programming of developmental genome rearrangements in Paramecium tetraurelia. Mol Cell Biol. 2004;24: 7370–7379. 10.1128/MCB.24.17.7370–7379.2004 15314149 PMC506981

[46] Bétermier M. Large-scale genome remodelling by the developmentally programmed elimination of germ line sequences in the ciliate Paramecium. Res Microbiol. 2004;155: 399–408. 10.1016/j.resmic.2004.01.017 15207872

[47] Marmignon A, Bischerour J, Silve S, Fojcik C, Dubois E, Arnaiz A, et al Ku-mediated coupling of DNA cleavage and repair during programmed genome rearrangements in the ciliate Paramecium tetraurelia. PLoS Genet. 2014;10: e1004552 10.1371/journal.pgen.1004552 25166013 PMC4148214

[48] Swart EC, Wilkes CD, Sandoval PY, Arambasic M, Sperling L, Nowacki M. Genome-wide analysis of genetic and epigenetic control of programmed DNA deletion. Nucleic Acids Res. 2014; 10.1093/nar/gku619 PMC4132734 25016527

[49] Juliano C, Wang J, Lin H. Uniting germline and stem cells: the function of Piwi proteins and the piRNA pathway in diverse organisms. Annu Rev Genet. 2011;45: 447–469. 10.1146/annurev-genet-110410-132541 21942366 PMC3832951

[50] Dang Y, Li L, Guo W, Xue Z, Liu Y. Convergent transcription induces dynamic DNA methylation at disiRNA loci. PLoS Genet. 2013;9: e1003761 10.1371/journal.pgen.1003761 24039604 PMC3764098

[51] Xu Z, Zan H, Pone EJ, Mai T, Casali P. Immunoglobulin class-switch DNA recombination: induction, targeting and beyond. Nat Rev Immunol. 2012;12: 517–531. 10.1038/nri3216 22728528 PMC3545482

[52] Desiderio S. Temporal and spatial regulatory functions of the V(D)J recombinase. Semin Immunol. 2010;22: 362–369. 10.1016/j.smim.2010.09.001 21036059

[53] Kapusta A, Matsuda A, Marmignon A, Ku M, Silve A, Meyer E, et al Highly Precise and Developmentally Programmed Genome Assembly in Paramecium Requires Ligase IV-Dependent End Joining. PLoS Genet. 2011;7 10.1371/journal.pgen.1002049 PMC3077386 21533177

[54] Sun C, Wyngaard G, Walton DB, Wichman HA, Mueller RL. Billions of basepairs of recently expanded, repetitive sequences are eliminated from the somatic genome during copepod development. BMC Genomics. 2014;15: 186 10.1186/1471-2164-15-186 24618421 PMC4029161

[55] Fish RN, Kane CM. Promoting elongation with transcript cleavage stimulatory factors. Biochim Biophys Acta. 2002;1577: 287–307. 12213659 10.1016/s0167-4781(02)00459-1

[56] Labhart P, Morgan GT. Identification of novel genes encoding transcription elongation factor TFIIS (TCEA) in vertebrates: conservation of three distinct TFIIS isoforms in frog, mouse, and human. Genomics. 1998;52: 278–288. 10.1006/geno.1998.5449 9790746

[57] Dehal P, Boore JL. Two rounds of whole genome duplication in the ancestral vertebrate. PLoS Biol. 2005;3 10.1371/journal.pbio.0030314 PMC1197285 16128622

[58] Uzureau P, Daniels JP, Walgraffe D, Wickstead B, Pays E, Gull K, et al Identification and characterization of two trypanosome TFIIS proteins exhibiting particular domain architectures and differential nuclear localizations. Mol Microbiol. 2008;69: 1121–1136. 10.1111/j.1365-2958.2008.06348.x 18627464 PMC2610381

[59] Ghosh S, Barrett DM, Klobutcher LA. The Euplotes crassus conjugation-specific conN1 gene encodes a transcription elongation factor TFIIS-like protein. J Eukaryot Microbiol. 2001;48: 218–220. 12095110 10.1111/j.1550-7408.2001.tb00305.x

[60] Miao W, Xiong J, Bowen J, Wang W, Liu Y, Braguinets O, et al Microarray analyses of gene expression during the Tetrahymena thermophila life cycle. PLoS ONE. 2009;4: e4429 10.1371/journal.pone.0004429 19204800 PMC2636879

[61] Ito T, Arimitsu N, Takeuchi M, Kawamura N, Nagata M, Saso K, et al Transcription elongation factor S-II is required for definitive hematopoiesis. Mol Cell Biol. 2006;26: 3194–3203. 10.1128/MCB.26.8.3194–3203.2006 16581793 PMC1446961

[62] Park KS, Cha Y, Kim CH, Ahn HJ, Kim D, Ko S, et al Transcription elongation factor Tcea3 regulates the pluripotent differentiation potential of mouse embryonic stem cells via the Lefty1-Nodal-Smad2 pathway. Stem Cells. 2013;31: 282–292. 10.1002/stem.1284 23169579 PMC3572291

[63] Fatica A, Bozzoni I. Long non-coding RNAs: new players in cell differentiation and development. Nat Rev Genet. 2014;15: 7–21. 10.1038/nrg3606 24296535

[64] Gratias A, Bétermier M. Processing of double-strand breaks is involved in the precise excision of paramecium internal eliminated sequences. Mol Cell Biol. 2003;23: 7152–7162. 14517286 10.1128/MCB.23.20.7152-7162.2003 PMC230320

[65] Gratias A, Bétermier M. Developmentally programmed excision of internal DNA sequences in Paramecium aurelia. Biochimie. 2001;83: 1009–1022. 11879729 10.1016/s0300-9084(01)01349-9

[66] Beisson J, Bétermier M, Bré MH, Cohen J, Duharcourt S, Duret L, et al Paramecium tetraurelia: the renaissance of an early unicellular model. Cold Spring Harb Protoc. 2010;2010: 10.1101/pdb.emo140 20150105

[67] Nelson MD, Fitch DH. Overlap extension PCR: an efficient method for transgene construction. Methods Mol Biol. 2011;772: 459–470. 10.1007/978-1-61779-228-1_27 22065455

[68] Timmons L, Fire A. Specific interference by ingested dsRNA. Nature. 1998;395: 854–854. 10.1038/27579 9804418

[69] Gogendeau D, Klotz C, Arnaiz O, Malinowska A, Dadlez M, de Loubresse NG, et al Functional diversification of centrins and cell morphological complexity. J Cell Sci. 2008;121: 65–74. 10.1242/jcs.019414 18057024

[70] Haacke B, Plattner H. Synchronous exocytosis in Paramecium cells. III. Rearrangement of membranes and membrane-associated structural elements after exocytosis performance. Exp Cell Res. 1984;151: 21–28. 6698118 10.1016/0014-4827(84)90352-5

[71] Galvani A, Sperling L. Transgene-mediated post-transcriptional gene silencing is inhibited by 3’ non-coding sequences in Paramecium. Nucleic Acids Res. 2001;29: 4387–4394. 11691926 10.1093/nar/29.21.4387 PMC60190

[72] Li H, Durbin R. Fast and accurate short read alignment with Burrows-Wheeler transform. Bioinformatics. 2009;25: 1754–1760. 10.1093/bioinformatics/btp324 19451168 PMC2705234

[73] Li H, Handsaker B, Wysoker A, Fennell T, Ruan J, Homer N, et al The Sequence Alignment/Map format and SAMtools. Bioinformatics. 2009;25: 2078–2079. 10.1093/bioinformatics/btp352 19505943 PMC2723002

[74] Dorai-Raj S. binom: Binomial Confidence Intervals for Several Parameterizations. [Internet]. 2009. Available: http://cran.r-project.org/web/packages/binom.

[75] Benjamini Y, Hochberg Y. Controlling the False Discovery Rate: A Practical and Powerful Approach to Multiple Testing. Journal of the Royal Statistical Society Series B (Methodological). 1995; Vol. 57: 289–300.

[76] Tamura K, Dudley J, Nei M, Kumar S. MEGA4: Molecular Evolutionary Genetics Analysis (MEGA) software version 4.0. Mol Biol Evol. 2007;24: 1596–1599. 17488738 10.1093/molbev/msm092

[77] Notredame C, Higgins DG, Heringa J. T-Coffee: A novel method for fast and accurate multiple sequence alignment. J Mol Biol. 2000;302: 205–217. 10.1006/jmbi.2000.4042 10964570

[78] Simossis VA, Heringa J. PRALINE: a multiple sequence alignment toolbox that integrates homology-extended and secondary structure information. Nucleic Acids Res. 2005;33: 289–294. 10.1093/nar/gki390 15980472 PMC1160151

[79] Combet C, Blanchet C, Geourjon C, Deléage G. NPS@: network protein sequence analysis. Trends Biochem Sci. 2000;25: 147–150. 10694887 10.1016/s0968-0004(99)01540-6

