# Supplementary material for: TFIIS-Dependent Non-coding Transcription Regulates Developmental Genome Rearrangements
Source: PLoS Genet. 2015 Jul 15;11(7):e1005383. doi: 10.1371/journal.pgen.1005383 (PMC4503560; doi:10.1371/journal.pgen.1005383)
Supplement: S1 Fig — Full protein sequences were aligned using T-Coffee [77] with default parameters and corrected manually. The alignment was colored using Boxshade at http://www.ch.embnet.org/software/BOX_form.html (grey: similar residues; black: identical residues; fraction of aligned residues that must agree for shading: 0.4). Only regions encompassing the conserved TFIIS domains are shown, since the region between domains I and II did not give significant alignment. The structural annotation below the alignment (structural features of the protein represented by filled rectangles) is based on the structure of S. cerevisiae Dst1p [21,32]. The Zn finger-forming conserved cystein residues and the DE dipeptide are highlighted in green and pink, respectively. The secondary structure prediction for P. tetraurelia TFIIS proteins was run using PSIPRED from the PRALINE package [78]: red and yellow open rectangles indicate the prediction of alpha helices in domains I and II or in the linker region, respectively. Blue open rectangles designate predicted beta-strands in domain III. Similar prediction results were obtained using NPS@ [79]. Abbreviations and accession numbers are as follows: Pt: Paramecium tetraurelia—accession numbers as in the legend of Fig 1A; Tt: Tetrahymena thermophila TFIIS—XP_001032085.3; Ot: Oxytricha trifallax 22233_0_g55—Contig22233_0_g55(protein), 1015_0_g5—Contig1015_0_g5(protein), 14486_0_g34—Contig14486_0_g34(protein) (OxyDB); Mc: Moneuplotes crassus conN1—AAG00939; Im: Ichthyophthirius multifiliis—IMG5_116810 (IchDB); Lm: Leishmania major Lm-TFIIS1-1—CAJ04034, Lm-TFIIS2-1—CAJ06790; Tb: Trypanosoma brucei TFIIS1—XP_828571, TFIIS2-1—XP_951597; Hs: Homo sapiens TCEA1—NP_006747, TCEA2—NP_003186, TCEA3—NP_003187; At: Arabidopsis thaliana TFIIS—NP_181390; Ce: Caenorhabditis elegans TFIIS—NP_495941; Dm: Drosophila melanogaster TFIIS—NP_476967.1, CG8117—NP_573049.2; Sp: Schizosaccharomyces pombe tfs1—CAC19733; Sc: Saccharomyces cerevisiae Dst1—NP_011472.1. (PDF) [file pgen.1005383.s001.pdf]

Figure S1

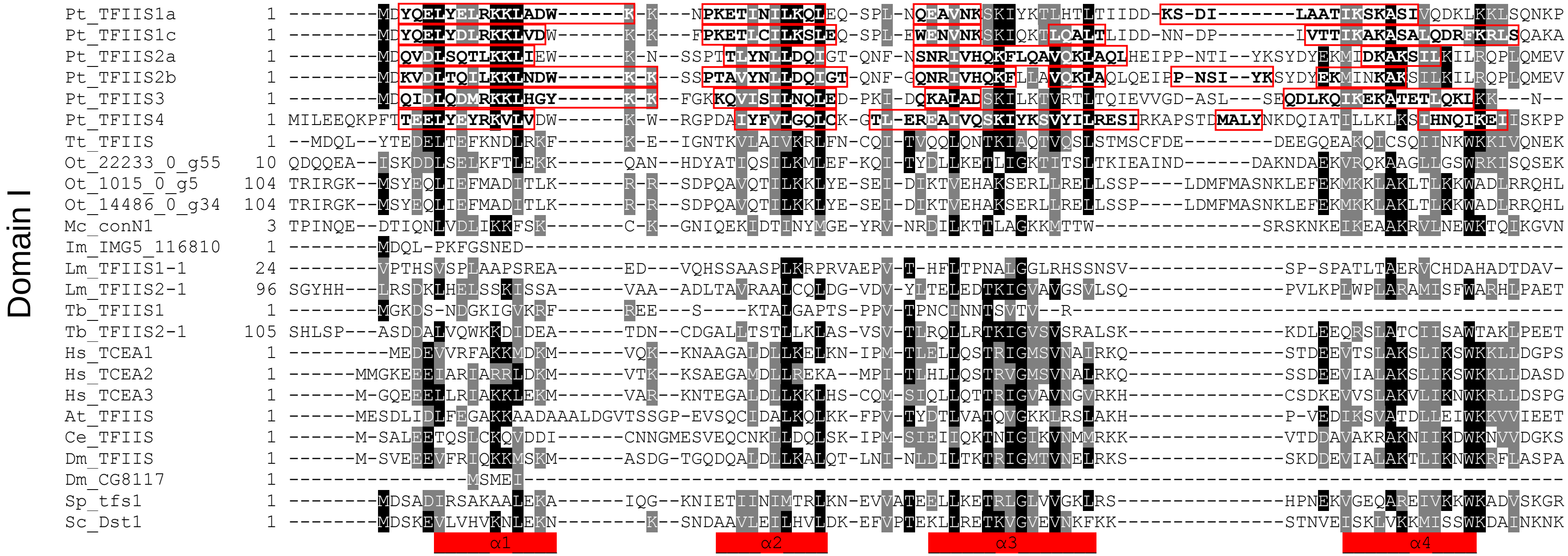
Domain I
Pt_TFIIS1a 1 ---------MDYQELYELRKKLADW------K-K---NPKETINILKQLEQ-SPL-NQEAVNKSKIYKTLHTLTIIDD-KS-DI-------LAATIKSKASIVQDKLKKLSQNKP
Pt_TFIIS1c 1 ---------MDYQELYDLRKKLVDW------K-K---FPKETLCILKSLEQ-SPL-EWENVNKSKIQKTLQALTLIDD-NN-DP-------IVTTIKAKASALQDRFKRLSQAKA
Pt_TFIIS2a 1 ---------MDQVDLSQTLKKLIEW------K-N--SSPTTLYNLLDQIGT-QNF-NSNRIVHQKFLQAVQKLAQLHEIPP-NTI--YKSYDYEKMIDKAKSILKILRQPLQMEV
Pt_TFIIS2b 1 ---------MDKVDLTQILKKLNDW------K-K--SSPTAVYNLLDQIGT-QNF-GQNRIVHQKFLLAVQKLAQLQEIPP-NSI--YKSYDYEKMINKAKSILKILRQPLQMEV
Pt_TFIIS3 1 ---------MDQIDLQDMRKKLHGY------K-K--FGKKQVISILNQLED-PKI-DQKALADSKILKTVRTLTQIEVVGD-ASL---SEQDLKQIKEKATETLQKLKK---N--
Pt_TFIIS4 1 MILEEQKPFTTEELYEYRKVLVDW------K-W--RGPDAIYFVLGQLCK-GTL-EREAIVQSKIYKSVYILRESIRKAPSTDMALYNKDQIATILLKLKSIHNQIKEIISKPP
Tt_TFIIS 1 --MDQL--YTEDELTEFKNDLRKF------K-E--IGNTKVLAIVKRLFN-CQI-TVQQLQNTKIAQTVQSLSTMSCFDE--------DEEGQEAKQICSQIINKWKKIVQNEK
Ot_22233_0_g55 10 QDQQEA--ISKDDLSELKFTLEKK------QAN--HDYATIQSILKMLEF-KQI-TYDLLKETLIGKTITSLTKIEAIND------DAKNDAEKVRQKAAGLLGSWRKISQSEK
Ot_1015_0_g5 104 TRIRGK--MSYEQLIEFMADITLK------R-R--SDPQAVQTILKKLYE-SEI-DIKTVEHAKSERLLRELLSSP----LDMFMASNKLEFEKMKKLAKLTLKKWADLRRQHL
Ot_14486_0_g34 104 TRIRGK--MSYEQLIEFMADITLK------R-R--SDPQAVQTILKKLYE-SEI-DIKTVEHAKSERLLRELLSSP----LDMFMASNKLEFEKMKKLAKLTLKKWADLRRQHL
Mc_conN1 3 TPINQE--DTIQNLVDLIKKFSK-------C-K--GNIQEKIDTINYMGE-YRV-NRDILKTTLAGKKMTTW---------------SRSKNKEIKEAAKRVLNEWKTQIKGVN
Im_IMG5_116810 1 ---------MDQL-PKFGSNED--
Lm_TFIIS1-1 24 ---------VPTHSVSPLAAPSREA------ED---VQHSSAASPLKRPRVAEPV-T-HFLTPNALGGLRHSSNSV--------------SP-SPATLTAERVCHDAHADTDAV-
Lm_TFIIS2-1 96 SGYHH---LRSDKLHELSSKISSA------VAA--ADLTAVRAALCQLDG-VDV-YLTELEDTKIGVAVGSVLSQ--------------PVLKPLWPLARAMISFWARHLPAET
Tb_TFIIS1 1 ---------MGKDS-NDGKIGVKRF------REE---S----KTALGAPTS-PPV-TPNCINNTSVTV--R---
Tb_TFIIS2-1 105 SHLSP---ASDDALVQWKKDIDEA------TDN--CDGALLTSTLLKLAS-VSV-TLRQLLRTKIGVSVSRALSK--------------KDLEEQRSLATCIISAWTAKLPEET
Hs_TCEA1 1 ----------MEDEVVRFAKKMDKM------VQK--KNAAGALDLLKELKN-IPM-TLELLQSTRIGMSVNAIRKQ--------------STDEEVTSLAKSLIKSWKKLLDGPS
Hs_TCEA2 1 ------MMGKEEEIARIARRLDKM------VTK--KSAEGAMDLLREKA--MPI-TLHLLQSTRVGMSVNALRKQ--------------SSDEEVIALAKSLIKSWKKLLDASD
Hs_TCEA3 1 ------M-GQEEELLRIAKKLEKM------VAR--KNTEGALDLLKKLHS-CQM-SIQLLQTTRIGVAVNGVRKH--------------CSDKEVVSLAKVLIKNWKRLLDSPG
At_TFIIS 1 ------MESDLIDLFEGAKKAADAAALDGVTSSGP-EVSQCIDALKQLKK-FPV-TYDTLVATQVGKKLRSLAKH--------------P-VEDIKSVATDLLEIWKKVVIEET
Ce_TFIIS 1 ------M-SALEETQSLCKQVDDI------CNNGMESVEQCNKLLDQLSK-IPM-SIEIIQKTNIGIKVNMMRKK--------------VTDDAVAKRAKNIIKDWKNVVDGKS
Dm_TFIIS 1 ------M-SVEEEVFRIQKKMSKM------ASDG-TGQDQALDLLKALQT-LNI-NLDILTKTRIGMTVNELRKS--------------SKDDEVIALAKTLIKNWKRFLASPA
Dm_CG8117 1 -----------------MSMEI--
Sp_tfs1 1 ---------MDSADIRSAKAALEKA------IQG--KNIETIINIMTRLKN-EVVATEELLKETRLGLVVGKLRS--------------HPNEKVGEQAREIVKKWKADVSKGR
Sc_Dst1 1 ---------MDSKEVLVHVKNLEKN--------K--SNDAAVLEILHVLDK-EFVPTEKLLRETKVGVEVNKFKK--------------STNVEISKLVKKMISSWKDAINKNK
α1
α2
α3
α4

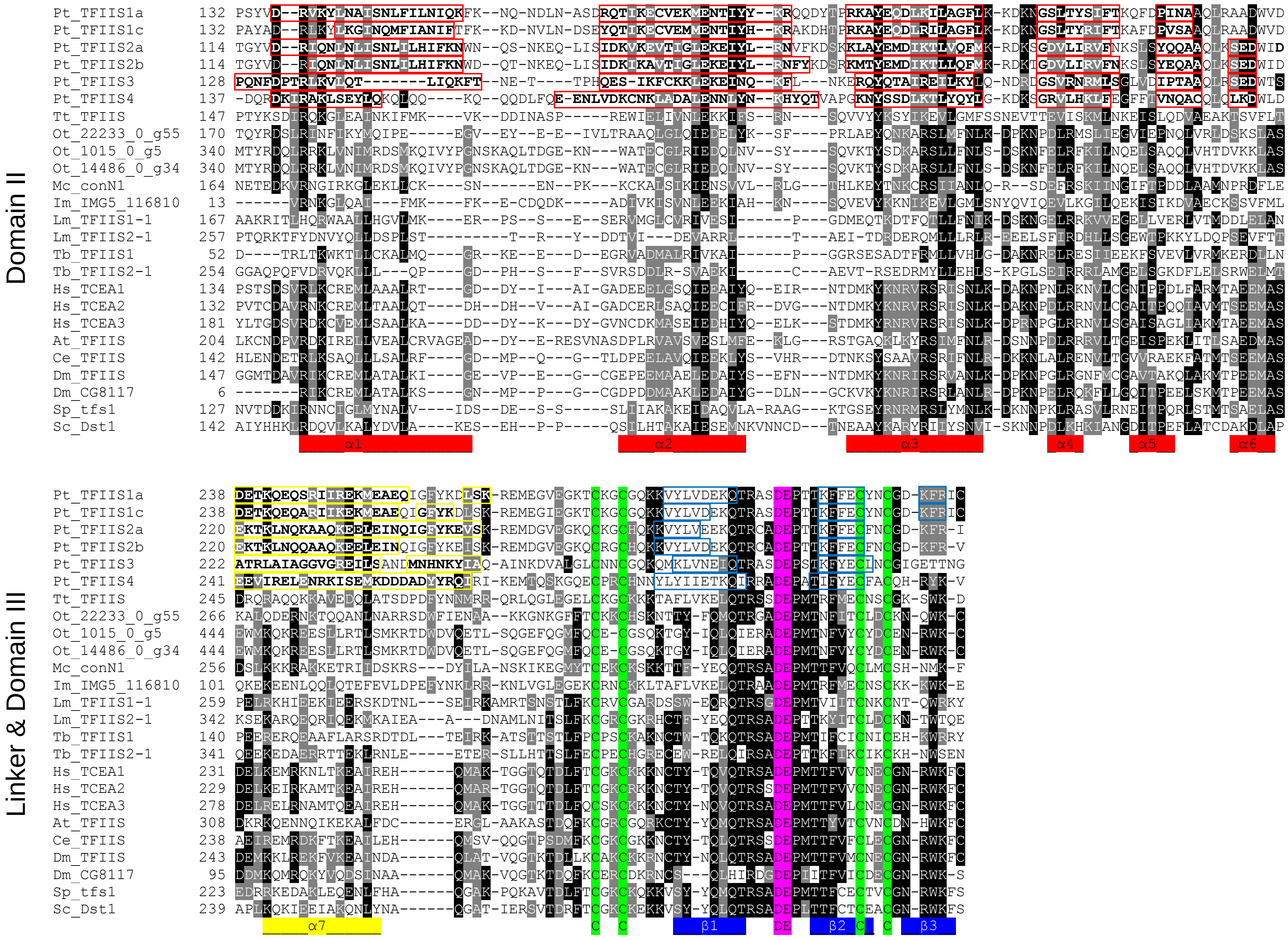
Domain II
Linker & Domain III
Pt_TFIIS1a
Pt_TFIIS1c
Pt_TFIIS2a
Pt_TFIIS2b
Pt_TFIIS3
Pt_TFIIS4
Tt_TFIIS
Ot_22233_0_g55
Ot_1015_0_g5
Ot_14486_0_g34
Mc_conN1
Im_IMG5_116810
Lm_TFIIS1-1
Lm_TFIIS2-1
Tb_TFIIS1
Tb_TFIIS2-1
Hs_TCEA1
Hs_TCEA2
Hs_TCEA3
At_TFIIS
Ce_TFIIS
Dm_TFIIS
Dm_CG8117
Sp_tfs1
Sc_Dst1
α1
α2
α3
α4
α5
α6
α7
C
C
β1
DE
β2
C
C
β3
