# Supplementary figures and images for: TFIIS-Dependent Non-coding Transcription Regulates Developmental Genome Rearrangements
Source: PLoS Genet. 2015 Jul 15;11(7):e1005383. doi: 10.1371/journal.pgen.1005383 (PMC4503560; doi:10.1371/journal.pgen.1005383)

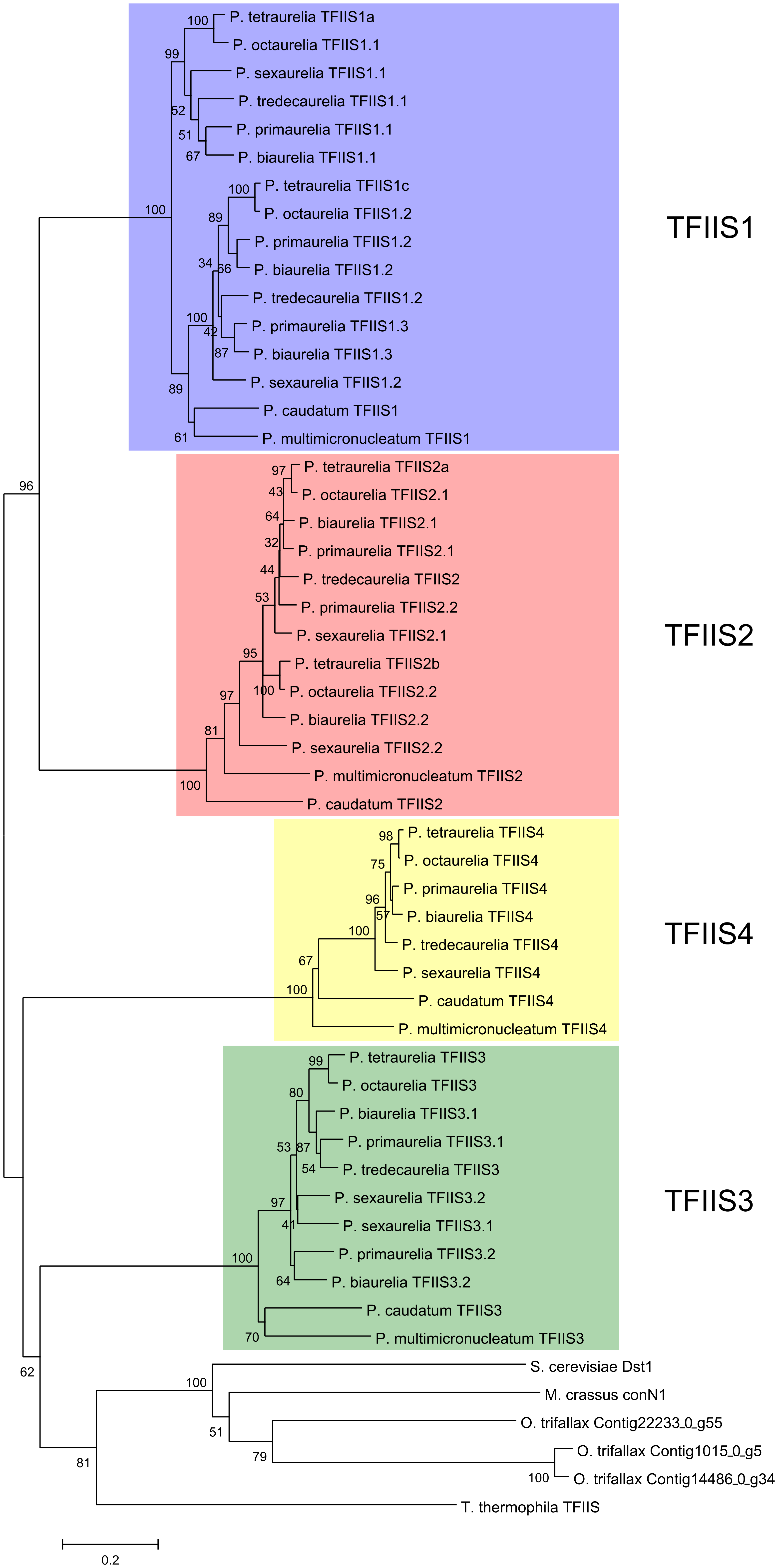

Supplement: S2 Fig — The evolutionary history was reconstructed as described in Fig 1 legend. (TIFF) [file pgen.1005383.s002.tiff]

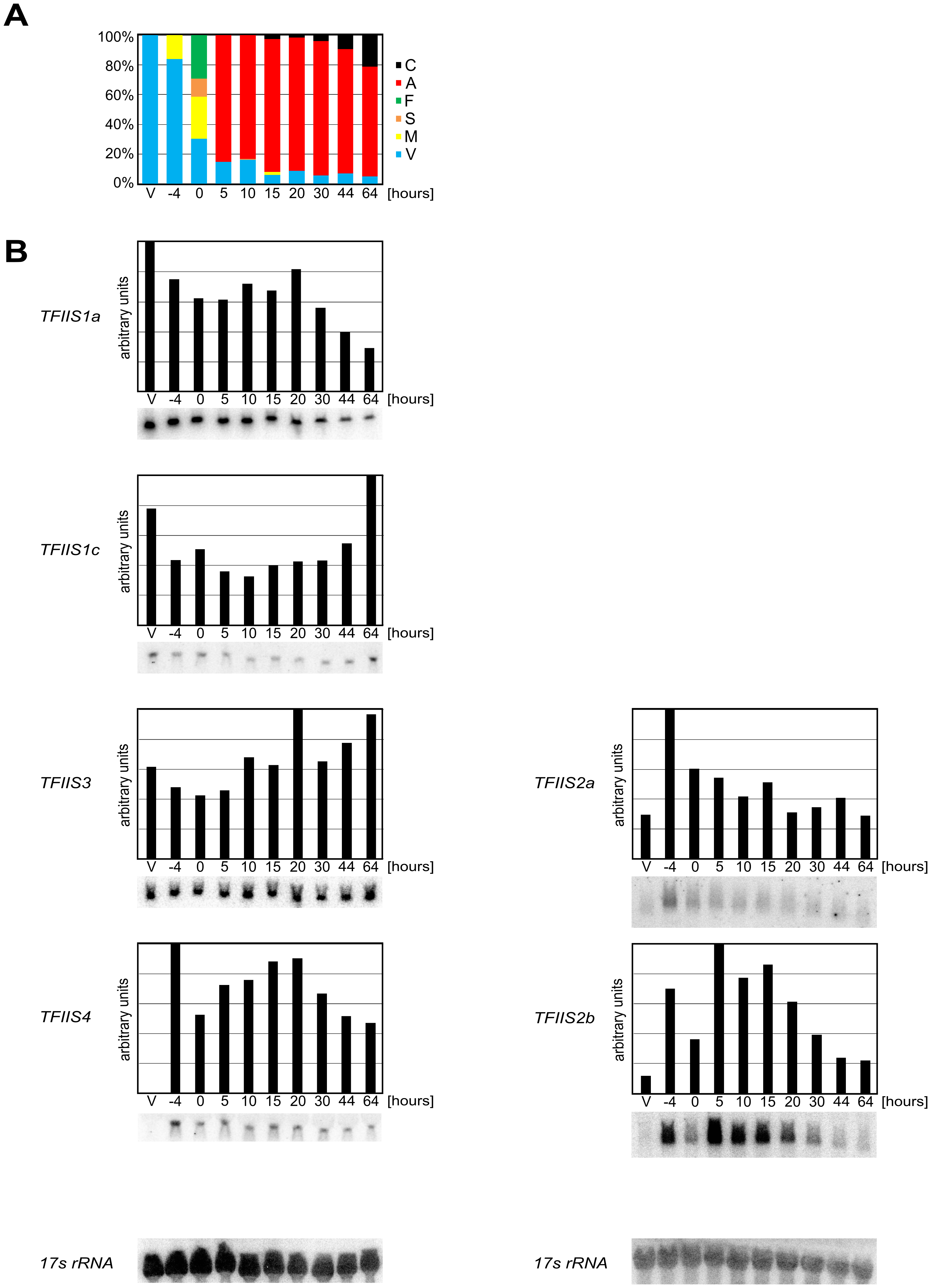

Supplement: S3 Fig — (A) Histograms show the progression of autogamy in strain 51new mt8 grown on standard K. pneumoniae medium. For each time-point (V: vegetative culture; -4: meiosis; 0: around 50% of cells with fragmented MAC; 5 to 64: 5 to 64 hours following time 0, respectively), cells were stained with DAPI to visualize old and new MACs. V: vegetative parental MAC; M: meiosis; S: skein formation; F: fragmented old MAC but no detectable developing new MACs; A: fragmented old MAC + 2 visible anlagen, C: post-karyonidal cells. (B) Northern blots and histograms showing the validation of expression profiles for each TFIIS gene. Two identical blots were used in parallel for the successive hybridization of individual 32P-labelled gene probes. Details for all hybridization probes are listed in S1 Table. Blot 1 was used for TFIIS1a, TFIIS1c, TFIIS3 and TFIIS4. Blot 2 was used for TFIIS2a and TFIIS2b. Hybridization signals were normalized using 17S rRNA. Hybridization of 32P-labelled 17S rDNA probe with each blot is shown at the bottom of the figure. (TIFF) [file pgen.1005383.s003.tiff]

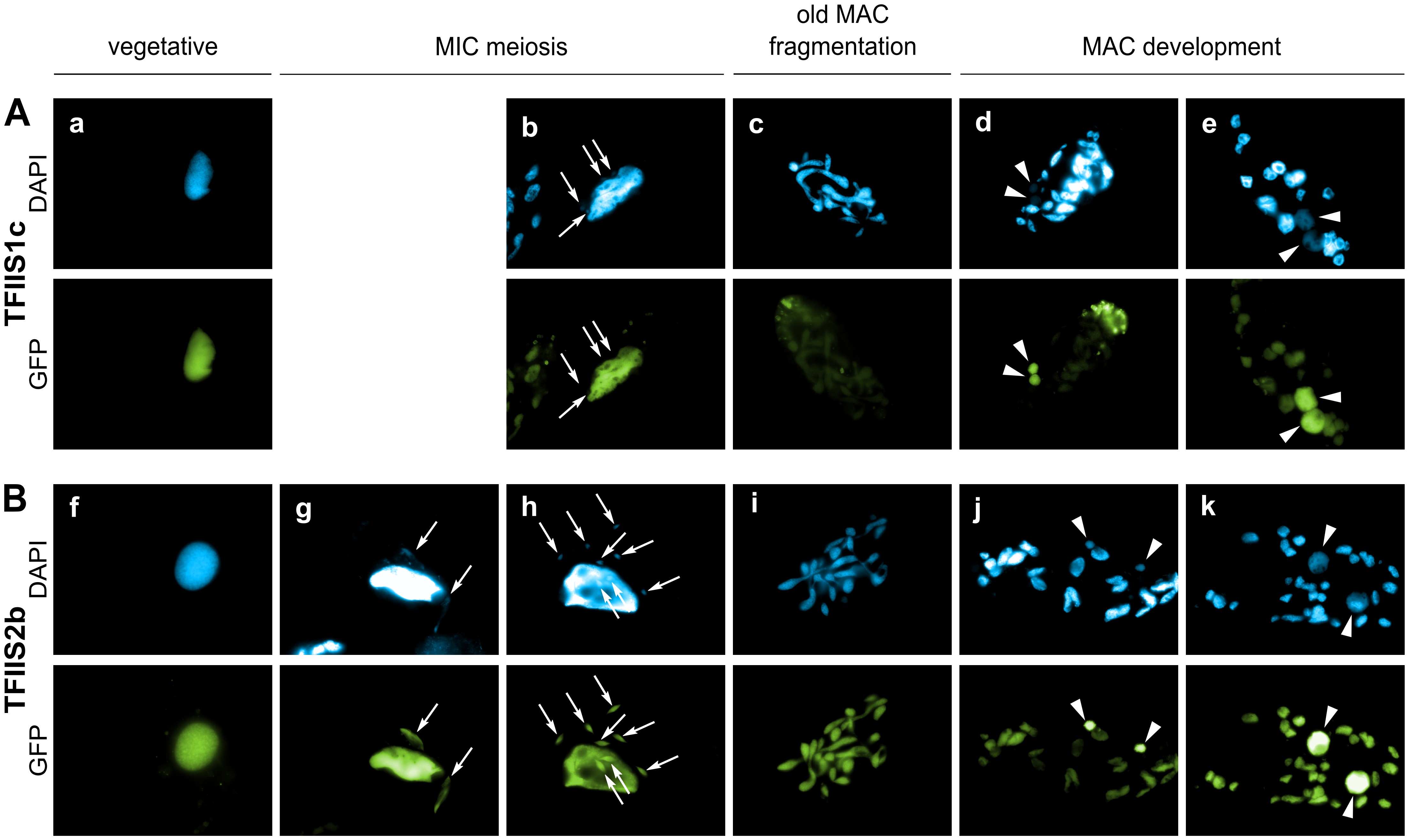

Supplement: S4 Fig — Panels a and f show vegetative cells. All other panels show successive stages of autogamy: panels b, g, h and i–meiosis: panel g–meiotic crescent stage; panel b–cell after meiosis I; panel h–cells with 8 haploid nuclei resulting from meiosis II; panels c and i–cells with fragmented old MAC; panels d and j–early MAC development; panels e and k–late MAC development. All arrows/arrowheads as in Fig 2. In panels c and d, the asterisks denotes additional fluorescent signal observed with the GFP filter due to the presence of crystals in the cytoplasm. (A) A GFP-TFIIS1c fusion localizes to old, then new MACs. (B) A GFP-TFIIS2b fusion localizes to old, then new MACs and is present in meiotic MICs. The GFP-TFIIS2b fusion shows a stronger signal in vegetative cells (panel f) than GFP-TFIIS2a—it might be explained by a higher copy number of the injected transgene, which may cause overexpression of the protein. (TIFF) [file pgen.1005383.s004.tiff]

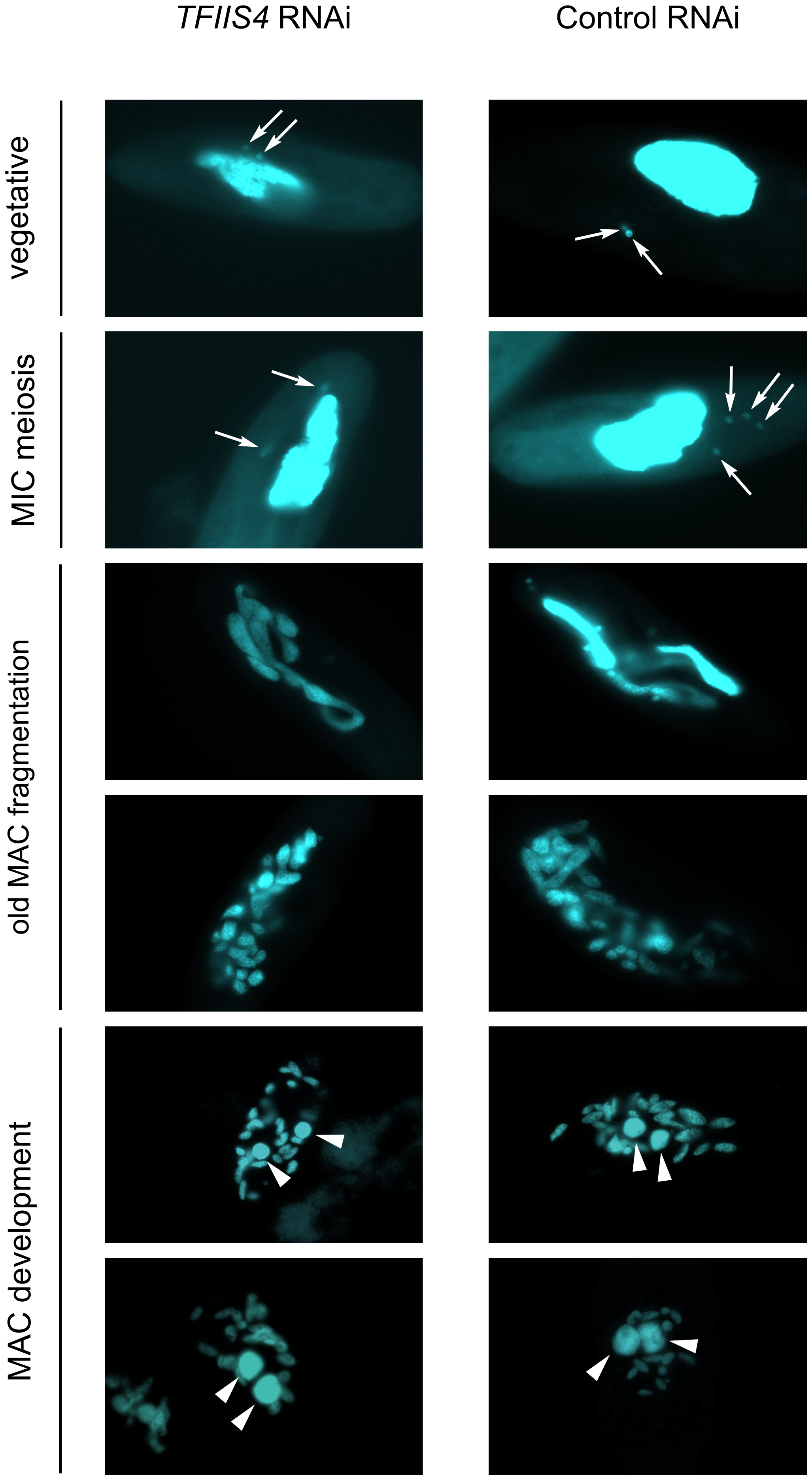

Supplement: S5 Fig — (TIFF) [file pgen.1005383.s005.tiff]

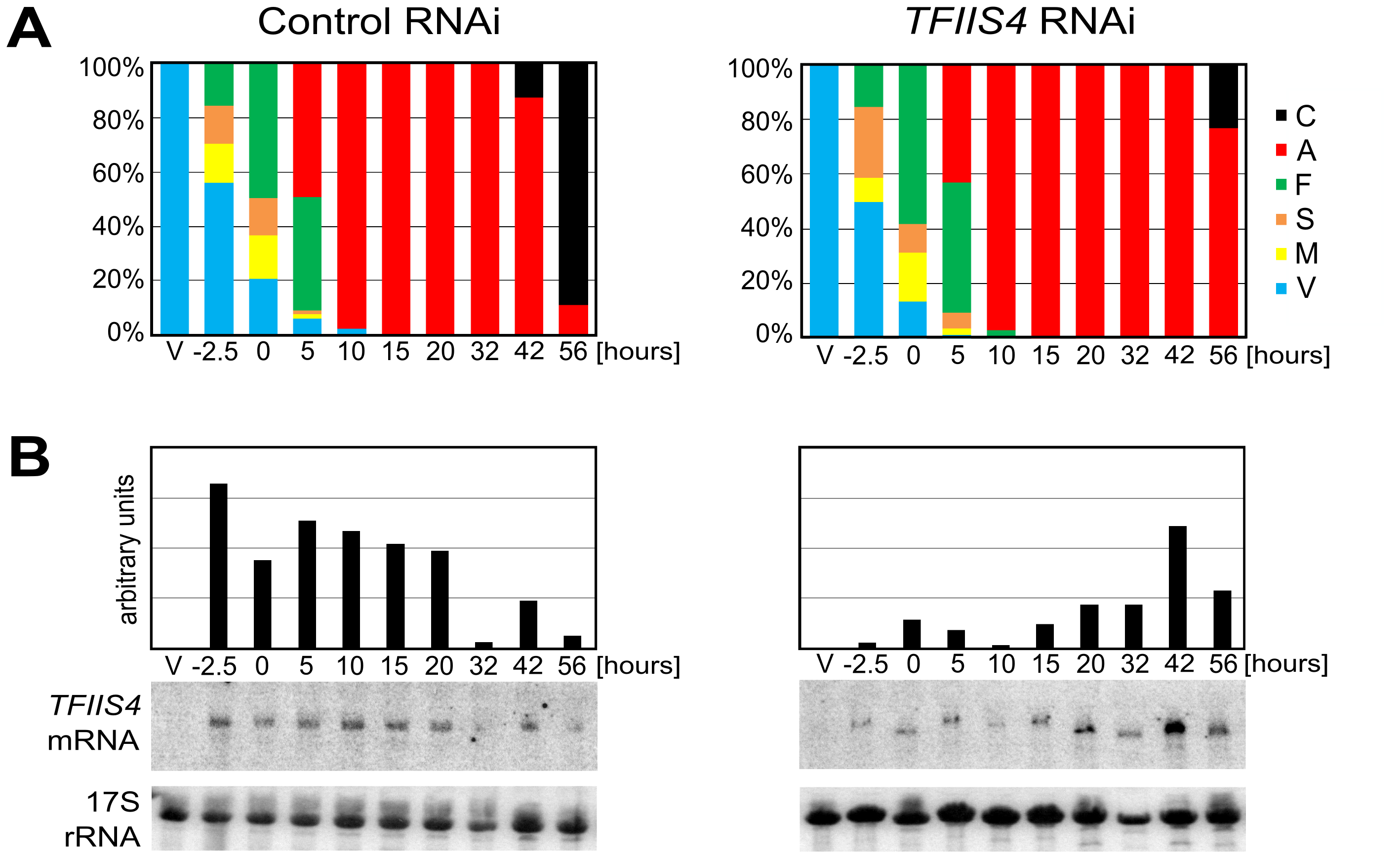

Supplement: S6 Fig — (A) Histograms show the progression of autogamy in strain 51mt8 ∆A ∆ND7. As a control we used cells silenced for the ICL7 unrelated gene. For each time-point (V: vegetative culture; -2.5: meiosis and early MAC fragmentation; 0: around 50% of cells with fragmented MAC; 5 to 56: 5 to 56 hours following time 0, respectively), cells were stained with DAPI to visualize old and new MACs. V: vegetative parental MAC; M: meiosis; S: skein formation; F: fragmented old MAC but no detectable developing new MACs; A: fragmented old MAC + 2 visible anlagen, C: post-karyonidal cells. (B) Northern blot validation of TFIIS4 silencing. The blot was hybridized sequentially with a TFIIS4 probe and 17S rRNA probe as a control of RNA loading. Histograms present expression of TFIIS4 during autogamy after normalization. In the latest time-points, transcripts corresponding to the gene are no longer efficiently down-regulated–probably due to lower amount of siRNA present in the cells after long starvation period. This may explain some residual survival in post-autogamous cells and delayed partial excision of some IESs. (TIFF) [file pgen.1005383.s006.tiff]

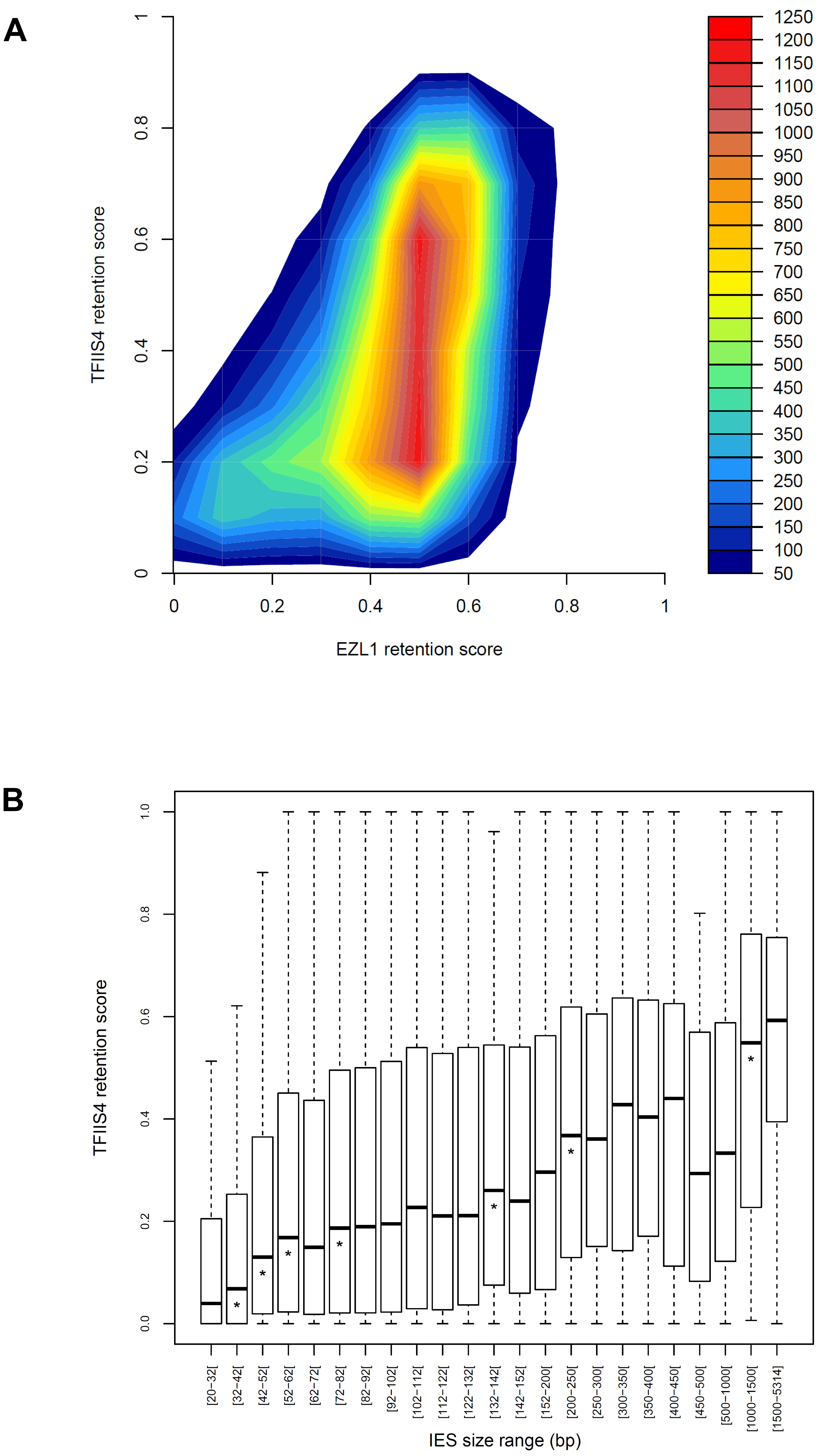

Supplement: S7 Fig — (A) The heatmap shows the relation between EZL1 retention score and TFIIS4 retention score for IESs that are significantly retained in TFIIS4 RNAi. The color represents the number of IESs according to the legend on the right. (B) Each group of IESs corresponds to a peak in the periodic IES size distribution [9]. The box plot displays the TFIIS4 IES retention score distribution for each group. The median retention score (horizontal line inside the box) and the first (top of box) and third (bottom of box) quartiles are shown. Stars beneath the median indicate that the retention score distribution of a given group is significantly different from the retention score distribution of the previous group according to a Mann-Whitney test. The median retention score significantly increases between the groups of small IESs (<82 bp), indicating that excision of the smallest IESs is mostly independent of TFIIS4 expression. For larger IESs the median increases slowly but the retention score distribution is significantly different only for IESs larger than 200 bp and, similarly to EZL1 and DCL2/3 [17,19], for the largest IESs (> 1 kb). (TIFF) [file pgen.1005383.s007.tiff]

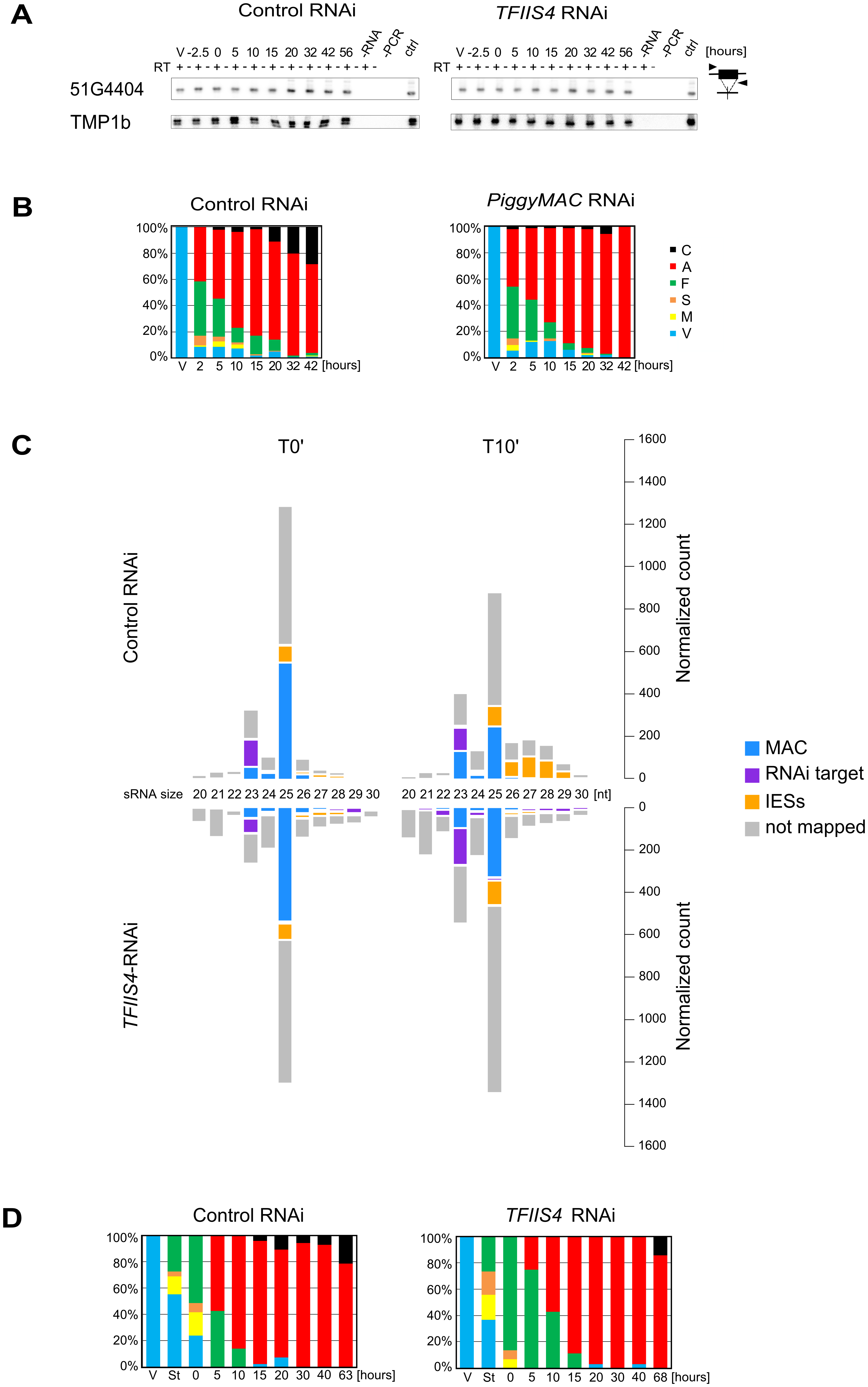

Supplement: S8 Fig — (A) Southern blot detection of G-gene transcripts obtained in RT-PCR reaction using primers located within the macronuclear sequences flanking IES 51G4404 (see S6 Fig, panel A for details about autogamy stages). PCR reactions were performed with the same set of first strand cDNA as used in IES+ transcript detection. PCR products (279 bp) correspond to IES-free (IES-) maternal transcripts. For each sample, the lane marked as “-” presents the control without reverse transcriptase. Lanes-RNA and-PCR are negative controls without RNA, ctrl corresponds to positive control performed on genomic DNA. (B) Histograms showing the progression of autogamy in a control culture (ND7-silenced cells) and in PiggyMac-silenced cells (strain 51new mt8) [47]. For details see legend to S6 Fig. (C) Histograms show normalized number of sRNAs that match to the Paramecium genome for the biological replicate of the experiment shown in Fig 7B. Details about autogamy stages are shown in S8 Fig, panel D. (D) Histograms show the progression of autogamy in strain 51mt8 ∆A ∆ND7 –biological replicate of the experiment used for most of the analysis. Cells were silenced for the TFIIS4 and ICL7 unrelated gene. For details see legend to S6 Fig. (TIFF) [file pgen.1005383.s008.tiff]

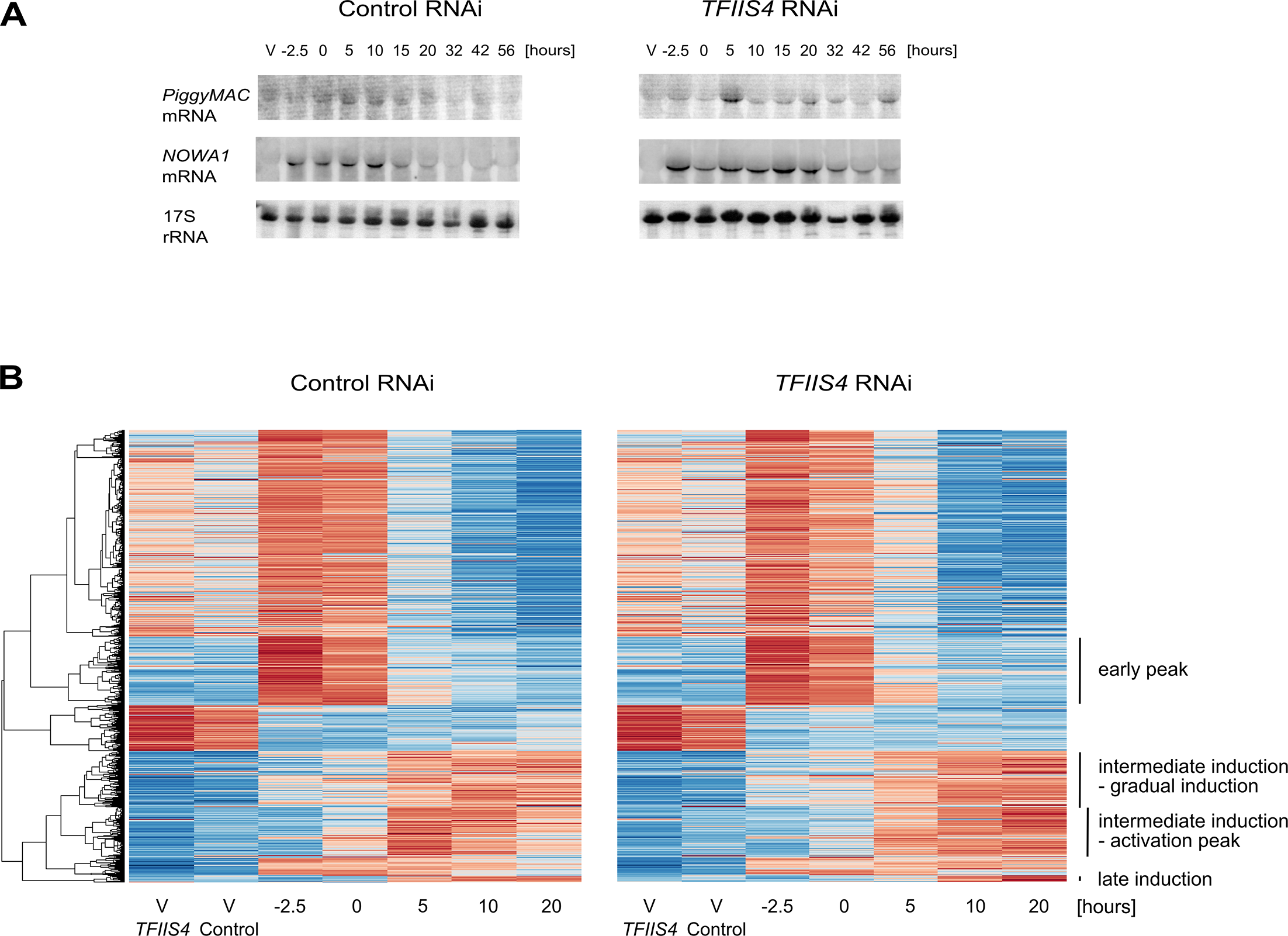

Supplement: S9 Fig — (A) Northern blot hybridization of RNA obtained in an autogamy time-course experiment (see S6 Fig), using PiggyMac and NOWA1/2 probes. 17S rRNA probe was used as a loading control. (B) Microarray hybridization data obtained using the same RNA samples as in panel A. Gene expression heatmap was plotted using the previously obtained hierarchical clusterization of the set of 2467 genes that are most differentially expressed during autogamy [28]. The plot displays the samples both for TFIIS4-silencing and the control (ICL7-silencing) as columns, and the genes as rows. The color code goes from dark blue for the lowest normalized expression level to dark red for the highest expression level. (TIFF) [file pgen.1005383.s009.tiff]

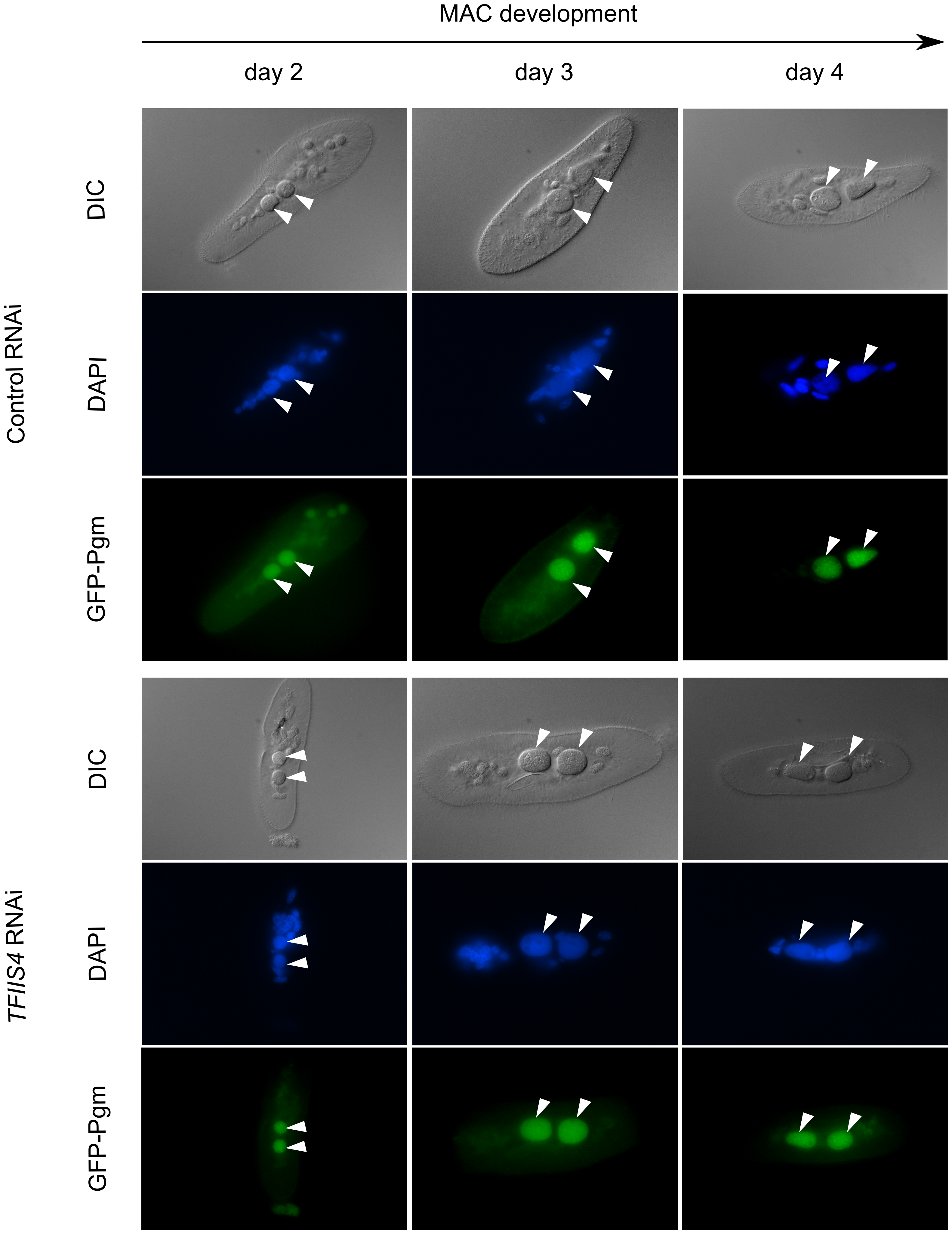

Supplement: S10 Fig — Previously described PiggyMac-GFP fusion construct was used in this study [10]. The efficiency of TFIIS4 silencing was confirmed by the 92% lethality observed in the sexual progeny. Control cells were silenced for the ICL7 gene. Vertical panels show cells at different stages of MAC development–after 2, 3 and 4 days of starvation, respectively. White arrowheads indicate new MACs. (TIFF) [file pgen.1005383.s010.tiff]

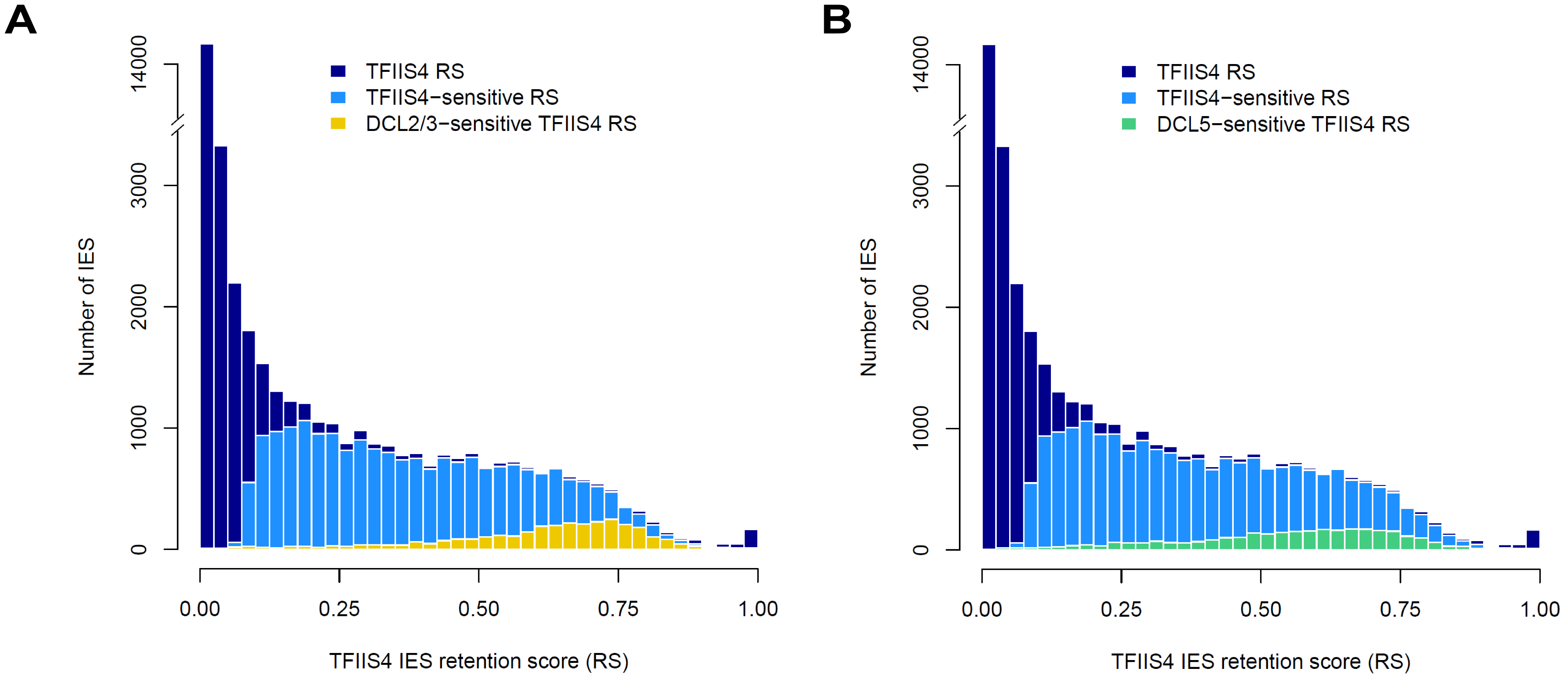

Supplement: S11 Fig — (A) Superimposed histogram of TFIIS4 retention scores for all IESs (dark blue) and IESs retained following DCL2/3 RNAi (yellow). Retention scores for IESs that are significantly retained in a TFIIS4 RNAi are in light blue. (B) As in (A) for Dcl5-dependent IESs (green). (TIFF) [file pgen.1005383.s011.tiff]
