# Supplementary material for: TFIIS-Dependent Non-coding Transcription Regulates Developmental Genome Rearrangements
Source: PLoS Genet. 2015 Jul 15;11(7):e1005383. doi: 10.1371/journal.pgen.1005383 (PMC4503560; doi:10.1371/journal.pgen.1005383)
Supplement: S1 Table — (PDF) [file pgen.1005383.s012.pdf]

Table S1. Coordinates of sequences used in this study as northern probes, inserts in GFP constructs and silencing constructs.

Gene Name	Gene ID	Acc. No. of scaffold	Coordinates of ORF	Coordinates of northern probe	Coordinates of fragment used for GFP-fusion construct	Coordinates of silencing insert
TFIIS1a	GSPATG00003556001	NW_001799630.1	272378..271880	272378..271880	272523..271301	272378..271411
TFIIS1c	GSPATG00008714001	NW_001799089.1	221560..222075	221560..222075	221309..222760	221560..222531
TFIIS2a	PTETG1100023001	NW_001798967.1	586182..585733	586239..586288	586383..585380	586306..585416
TFIIS2b	GSPATG00003298001	NW_001799619.1	624195..623735	624285..624334	624448..623339	624352..623462
TFIIS3	GSPATG00019582001	NW_001799522.1	172955..172457	172955..172457	173114..171918	172955..171979
TFIIS4	GSPATG00025792001	NW_001799642.1	151716..150602	150602..150859	151832..150423	150860..151702
