# Supplementary material for: TFIIS-Dependent Non-coding Transcription Regulates Developmental Genome Rearrangements
Source: PLoS Genet. 2015 Jul 15;11(7):e1005383. doi: 10.1371/journal.pgen.1005383 (PMC4503560; doi:10.1371/journal.pgen.1005383)
Supplement: S2 Table — (PDF) [file pgen.1005383.s013.pdf]

Table S2. Oligonucleotides used in the study.

Oligo name	Sequence (5’ to 3’)	Application
51A2591-18	AAGTGCAACCTGTGCTGATGCTCCCGATGA	PCR around IES 51A2591
51A2591-20	AGTTCCTTTGAAAGATGTGCAAGCTCCAGA	PCR around IES 51A2591
51A4578-3'	TGTAGTCTTAAAATCTTAGCATGTTGTACC	PCR around IES 51A4578
51A4578-5'	CACTGCAGTAAGTTGCAGTTCAATAACTGG	PCR around IES 51A4578
51A1835-5'	TAATGTATTGATAAGGCTTGCTCTACAGCC	PCR around IES 51A1835
51A1835-3'(3)	GTAGTACAAGATTTTTCGACACAAGTTGAG	PCR around IES 51A1835
51A4404-3'(2)	CCAGTTATTGAACTGCAACTTACTGCAGTG	PCR around IES 51A4404
51A4404-5'	TAAATGTTCAGCTTACAACGCAGCT	PCR around IES 51A4404
51G2832 up	GAGCAGGATGTACAAATACTGGTGG	PCR around IES 51G2832
51G2832 lo	AGCTGATTAGATAACAATACAACCAGTACC	PCR around IES 51G2832
51G18	ACTGTTGCTACACATTGTGCATATGTTACT	PCR around IES 51G4404
51G19	GCTGTAAGATTAACATTGAGCATGATCAAG	PCR around IES 51G4404
51A6649-12(2)	ACTGCACCTCTAACTTTAACAAGCGAAGCA	PCR around IES 51A6649
51A6649-3'	CAGCAGTACATCCAGCTCTCTAAGTTTAGC	PCR around IES 51A6649
51A-712-10	TTTGTCAAAAAGACATGTATCAAAATGCAG	PCR around IES 51A-712
51A-712-11	TAGAATACTAAGAGATTCAATACAACAAAC	PCR around IES 51A-712
51G1832 up	GCTATAACTCTTGAAGCTGCTTGTAATATG	PCR around IES 51G1832
51G1832 lo	TTGTCAATGAGCCATTAACAGTTGCTGGAT	PCR around IES 51G1832
sm19-1	CTAAGAAAATGAGCAAATAGTGGAATAAAT	PCR around IES sm19-576
sm19-4	GACAAGATCCTATATATTCATTTACATTTG	PCR around IES sm19-576
51A6435-5'(2)	TTCCAAGCCTTAGTCAATCAATCAGGATGTGTC	PCR around IES 51A6435
51A6435-3'(2)	TTTGTGAAATAACTAGCACAAGCTGCTTCG	PCR around IES 51A6435
PBl49-01	TAGAGGCATTGGAGCAGTGGGAACTATTAG	*PGM* hybridization probe
PBl49-02	AAGTTTCTCTAAGAAATTTGGCTTGTATGG	*PGM* hybridization probe
mit1	GGGGGCGGGCGGGCTCAAGTGCTGC	mitochondrial probe
mit2	TAGGCCGTCGTCTTTAAGAAGGAGT	mitochondrial probe
51G18	ACTGTTGCTACACATTGTGCATATGTTACT	PCR for detection of maternal transcripts
51G17	GATCAAGTCCAGTTCCTGTTATAGAACTAC	PCR for detection of maternal transcripts
51G15	TTAATGCTCATTCATCCAAATTAGCCTTTG	PCR for IES+ 51G4404 probe
51G06	CAGCCGGTATTATTAAGTAACTTTAAAAAG	PCR for IES+ 51G4404 probe/IES+ transcripts
51G05	GATGGTATTATAAATCGAATATTAATTCTG	PCR for detection of IES+ 51G4404 transcripts
51A6649-05	GCAAAACTCAAGACATTGTAGTTAATTTCC	PCR for IES+ 51A6649 probe
51A6649-02	ACAGGTTAAAAAAAATTTTTTTCTATTTTC	PCR for IES+ 51A6649 probe/IES+ transcripts
51A6649-13	TAATCATACTCCTAGATTAGAAG	PCR for detection of IES+ 51A6649 transcripts
51A4404pl	AAGTAATATATTTTTTAAAATATTTGGTTT	PCR for IES+ 51A4404probe

51A4404B	AGTATGGTAGGTTTAAGTTTTTTTTATG	PCR for IES+ 51A4404probe/IES+ transcripts
51A4404-A	ATTGTGGTGTTATTTTAAAAAGTAATATATT	PCR for detection of IES+ 51A4404 transcripts
T1b-3'	TTGAGTTGGGATTTGACATAATCGGTGAA	PCR for detection of mRNA of T1-b gene
T1b-5'(2)	TCTAATTAAACCAAGAACACGCTGAATTCC	PCR for detection of mRNA of T1-b gene
