# Supplementary material for: TFIIS-Dependent Non-coding Transcription Regulates Developmental Genome Rearrangements
Source: PLoS Genet. 2015 Jul 15;11(7):e1005383. doi: 10.1371/journal.pgen.1005383 (PMC4503560; doi:10.1371/journal.pgen.1005383)
Supplement: S3 Table — This table shows the sequence complexity of PGM, TFIIS4 and the control (wild-type genome) datasets, using the contigs assembled from the PGM dataset as a reference. Sequencing reads were mapped on the entire reference, and coverage of each contig was determined in RPKM (reads per kilobase (kb) of contig per million mapped reads in the library). We consider that a contig is covered if its coverage is above 2 RPKM. The “PGM” reference contains contigs larger than 1 kb and covered by the PGM dataset. The “PGM not Control” contains contigs larger than 1 kb, covered by the PGM dataset but not by the control dataset, representing the MIC restricted regions, not collinear with the MAC. Each column indicates sum of the lengths of contigs covered by the given dataset. (PDF) [file pgen.1005383.s014.pdf]

Table S3. Sequence complexity of control, PGM and TFIIS4 datasets.

Reference	PGM	TFIIS4	Control
PGM	88 996 911 bp<br>100.0%	84 382 513 bp<br>94.8%	76 088 297 bp<br>85.5%
PGM not Control	12 908 614 bp<br>100.0%	8 332 941 bp<br>64.5%	0<br>0.0%
